# phyloseq: An R Package for Reproducible Interactive Analysis and Graphics of Microbiome Census Data

**DOI:** 10.1371/journal.pone.0061217

**Published:** 2013-04-22

**Authors:** Paul J. McMurdie, Susan Holmes

**Affiliations:** Department of Statistics, Stanford University, Stanford, California, United States of America; The Roslin Institute, University of Edinburgh, United Kingdom

## Abstract

**Background:**

The analysis of microbial communities through DNA sequencing brings many challenges: the integration of different types of data with methods from ecology, genetics, phylogenetics, multivariate statistics, visualization and testing. With the increased breadth of experimental designs now being pursued, project-specific statistical analyses are often needed, and these analyses are often difficult (or impossible) for peer researchers to independently reproduce. The vast majority of the requisite tools for performing these analyses reproducibly are already implemented in R and its extensions (packages), but with limited support for high throughput microbiome census data.

**Results:**

Here we describe a software project, phyloseq, dedicated to the object-oriented representation and analysis of microbiome census data in R. It supports importing data from a variety of common formats, as well as many analysis techniques. These include calibration, filtering, subsetting, agglomeration, multi-table comparisons, diversity analysis, parallelized Fast UniFrac, ordination methods, and production of publication-quality graphics; all in a manner that is easy to document, share, and modify. We show how to apply functions from other R packages to phyloseq-represented data, illustrating the availability of a large number of open source analysis techniques. We discuss the use of phyloseq with tools for reproducible research, a practice common in other fields but still rare in the analysis of highly parallel microbiome census data. We have made available all of the materials necessary to completely reproduce the analysis and figures included in this article, an example of best practices for reproducible research.

**Conclusions:**

The phyloseq project for R is a new open-source software package, freely available on the web from both GitHub and Bioconductor.

## Introduction

### Phylogenetic Sequencing

High-throughput (HT) DNA sequencing [1] is allowing major advances in microbial ecology studies [2], where our understanding of the presence and abundance of microbial species relies heavily on the observation of their nucleic acids in a “culture independent” manner [3]. This nucleic acid sequencing based census of the inhabitants of microbiome samples is very often now accompanied with other experimental observations (e.g. clinical, environmental, metabolomic, etc.), in addition to phylogenetic tree reconstruction and/or taxonomic classification of the sequences. Here we refer to this as “phylogenetic sequencing” data if it can be usefully represented as a contingency table of taxonomic units and samples, and integrated with the other aforementioned data types. Importantly, this term – also the namesake of the software here described – is defined so as to not be specific to the method by which the phylogenetically relevant microbial census data was obtained, reflecting the intended level of data abstraction in the software. The following are two examples of common methods for producing phylogenetic sequencing data.

Barcoded [2] amplicon sequencing of dozens to hundreds of samples [4] is a method of phylogenetic sequencing of microbiomes, often targeting the small subunit ribosomal RNA (16S rRNA) gene [3], for which there are also convenient tools [5] and large reference databases [6]–[8]. The task of decoding the sample source of each sequence read by its barcode, followed by similarity clustering to define *operational taxonomic units* (OTUs, sometimes referred to as *taxa*) [9], [10] can be performed by publicly available packages/pipelines, including QIIME [11], mothur [12], and PANGEA [13]; as well as virtual machine (VM) and cloud-based solutions such as the RDP pipeline [7], Pyrotagger [14], CLoVR-16S [15], Genboree [16], QIIME EC2 image [17], n3phele [18], and MG-RAST [19].

**Figure 1:**
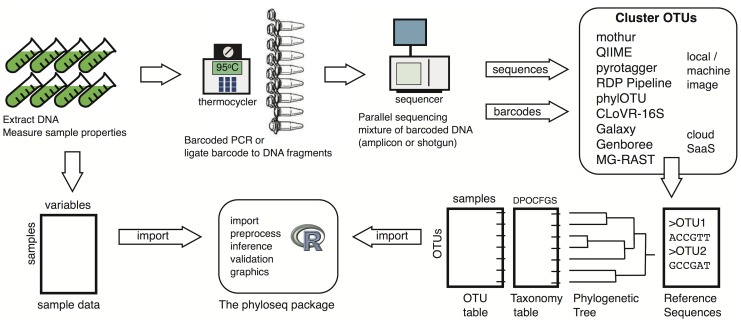
Example of a phylogenetic sequencing workflow. A diagram of an experimental and analysis workflow for amplicon or shotgun phylogenetic sequencing. The intended role for phyloseq is indicated.

An alternative experimental method is random “shotgun” sequencing [20], [21] of un-amplified metagenomic DNA [22], in which case OTU clustering and counting is based upon one or more detectable phylogenetic markers in the metagenomic sequence fragments, using tools such as phylOTU [23]. It is worth noting that bias from PCR amplification is avoided in this latter approach – at the expense of per-sequence efficiency [23] – and both methods are now commonly used for phylogenetic sequencing (Figure 1).

### The phyloseq Project

Many of the previously mentioned OTU-clustering applications also perform additional downstream analyses (File S1). However, typically an investigator must port the human-unreadable output data files to other software for additional processing and statistical analysis specific to the goals of the investigation. The powerful statistical, ecological, and graphics tools available in R [24] make it an attractive option for this post-clustering stage of analysis. While the computational efficiency of compiled languages like 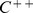 [25] make them appropriate for the expensive but well-defined requirements of the initial sequence-processing, the subsequent analysis is vaguely-defined and project specific; requiring instead a broad set of interactive calculations that is often less computationally expensive and for which R is well-suited [26]. The public repositories of open-source R extensions (“packages” or “libraries”) include many dedicated ecology and phylogenetic packages. For instance, there are several dozen packages listed in the CRAN Ecology Task View [27], as well as distory [28], phangorn [29], picante [30], and now phyloseq [31]. Furthermore, R includes infrastructure for documenting an analysis in such a way that it can be easily reproduced and modified by peers [32], [33].

In spite of all of these highly relevant tools, we recently described the lack of a satisfactory standard within *Bioconductor* [34] (or R generally) for importing the data files from the most popular OTU-clustering applications, or representing this data in a complete, integrated class [31]. One Bioconductor package, OTUbase [35], pursues some of these goals, but has no support for phylogenetic trees in its data class, nor support for importing data from popular/recent OTU-clustering output formats [35], [36] (File S1). We have proposed a new Bioconductor package, phyloseq (from “phylogenetic sequencing”), dedicated to the object-oriented representation and analysis of phylogenetic sequencing data in R [31], and supporting common OTU-clustering output formats like QIIME [11], mothur [12], the RDP-pipeline [7], Pyrotagger [14], and the biom-format [37].

In this article we describe the conceptual framework and toolbox of a substantially enhanced phyloseq codebase, including especially some advanced ordination and graphics capabilities. We further note that data imported by phyloseq is also accessible to analyses encoded by a large number of freely available R packages, in addition to the capabilities directly supported by phyloseq itself. We will end by discussing the notion of “reproducible research” in the context of phylogenetic sequencing data, and how phyloseq and R can be used in analyses that are more open and reproducible than those found in recent common practice.

## Methods

### phyloseq Project Key Features

The phyloseq package provides an object-oriented programming infrastructure that simplifies many of the common data management and preprocessing tasks required during analysis of phylogenetic sequencing data. This simplified syntax helps mitigate inconsistency errors and encourages interaction with the data during preprocessing. The phyloseq package also provides a set of powerful analysis and graphics functions, building upon related packages available in R and Bioconductor. It includes or supports some of the most commonly-needed ecology and phylogenetic tools, including a consistent interface for calculating ecological distances and performing dimensional reduction (ordination). The graphics functions allow users to interactively produce annotated publication-quality graphics in just one or two lines of code. The phyloseq package includes extensive documentation in the form of function- and package-level manuals embedded in the package's documentation interface and in a PDF version on Bioconductor [38], as well as extended reproducible examples on the phyloseq homepage [39], and open collaborative development on GitHub [40].

### Implementation

The phyloseq package adheres to the requirements for standard R packages set forth in the official “Writing R Extensions” manual [40]. It also satisfies additional requirements of the Bioconductor Repository [34], and uses a literate-programming framework based on structured in-source comments, called roxygen2 [41], for (re)building the R documentation (.Rd) files and the namespace specifications. The phyloseq package can be installed on any system on which R is supported, including Mac OS X, Windows, and most Linux distributions.

### Data Availability

R packages can include example data that is documented with the same help system as other package objects [58]. This data becomes available in the R session by invoking the data function after the package has been loaded. Unless otherwise noted, the examples provided in this manuscript use example data that is included in the phyloseq package.

### Data Infrastructure and Design

**Figure 2:**
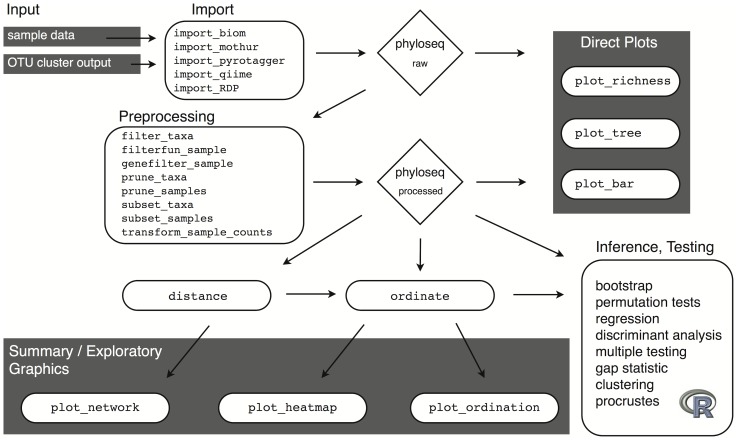
Analysis workflow using phyloseq. The workflow starts with the results of OTU clustering and independently-measured sample data (Input, top left), and ends at various analytic procedures available in R for inference and validation. In between are key functions for preprocessing and graphics. Rounded rectangles and diamond shapes represent functions and data objects, respectively, further described in Figure 3.

The phyloseq project includes an object-oriented class that integrates the heterogeneous components of OTU-clustered phylogenetic sequencing data. Although Bioconductor provides many utilities for efficient manipulation of DNA sequences, phyloseq does not currently re-implement any methods for DNA sequence decoding, processing, or OTU-clustering (Figure 1, File S1). Instead, phyloseq provides tools to read the output files of the most common OTU-clustering applications [7], [11], [12], [14], and represents this data in R as an instance of the main data class. This multi-component “experiment-level” class — named “phyloseq”, and referred to here as “the phyloseq-class” — is a key design feature of the phyloseq project, with subsequent user-accessible functions expecting to operate on an instance of this class as their sole or primary input data. These functions are described in detail in the phyloseq manual [7], and are part of a modular workflow summarized in Figure 2.

**Figure 3:**
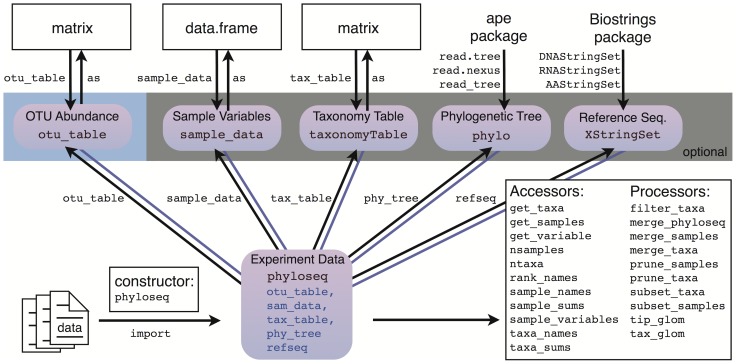
The “phyloseq” class. The phyloseq class is an experiment-level data storage class defined by the phyloseq package for representing phylogenetic sequencing data. Most functions in the phyloseq package expect an instance of this class as their primary argument. See the phyloseq manual [38] for a complete list of functions.

Figure 3 summarizes the structure of the phyloseq-class and its components. Each of the slots are empty (NULL) by default, although an instance missing an otu_table component is invalid. Tools in phyloseq that truncate dimensions of one component (that is, remove samples or OTUs) automatically propagate the change across all relevant components. In general, researchers only need to manipulate their “experiment-level” object, making data (pre)processing less prone to mistakes, and often simplifying analysis commands to just one data argument.

### Analysis Functions

Complementing the data infrastructure, the phyloseq package provides a set of functions that take a phyloseq object as the primary data, and performs an analysis and/or graphics task. Figure 2 summarizes the general workflow within phyloseq, and lists some of the main functions/tools.

Comparisons of the type and quantity of OTUs observed between microbiome samples (“beta diversity”) is often approached through the calculation of pairwise ecological distances [42], [43], and through dimensional reduction (ordination) methods. The phyloseq package provides a consistent interface for the most common approaches to distance calculations and ordination. This interface is also the foundation for the custom ordination and heatmap graphics functions described in the next subsection.

In phyloseq the interface for ecological distance calculations is a single function, distance, that takes a phyloseq object as its data argument as well as a character string indicating the distance method, with explicit support for more than 40 ecological distance methods. This includes a R -native, optionally-parallel implementation of Fast UniFrac [44] (both weighted [45] and unweighted [46]). The output is a “dist” class distance matrix (lower-triangle) appropriate for standard clustering analysis in core R (e.g. hclust), as well as certain dimensional reduction (ordination) methods.

The interface for performing ordination methods is also a single function, called ordinate, that takes a phyloseq object as its primary data argument and a character string indicating the desired ordination method. For example, the following would perform (unconstrained) correspondence analysis on the included “Global Patterns” dataset [47].
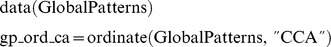



The ordinate function currently supports correspondence analysis (CA) [48], constrained correspondence analysis (CCA) [49], detrended correspondence analysis (DCA) [50], redundancy analysis (RDA) [51], principal components analysis (PCA) [52], double principle coordinates analysis (DPCoA) [53], multidimensional scaling (MDS, PCoA) [54], and non-metric multidimensional scaling (NMDS) [55]. For CA, CCA, DCA, RDA, and DPCoA, the ordination is based upon an evaluation of abundance values (in the case of DPCoA, the patristic distances between OTUs on the phylogenetic tree is also used), but not an ecological distance. For MDS and NMDS, the ordinate function requires a pre-calculated distance matrix (“dist” object) or the name of a supported ecological distance method. For example, PCoA/MDS can be calculated on an unweighted UniFrac distance matrix [46], using the following command:
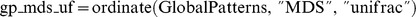



There are many combinations of approaches possible (even extending into time-series of table pairs), and the optimal approach depends on the goals of the experiment and characteristics of the data [56]. The phyloseq package also includes a specialized function for displaying ordination results in different ways, described in the following section.

### Specialized Graphics

One of the key features of the phyloseq package is a set of graphics functions custom-tailored for phylogenetic sequencing analysis, built using the ggplot2 package [57]. The ggplot2 package is an implementation of Wilkinson's *The Grammar of Graphics*, which provides an object-oriented description of analytical graphics that emphasizes the separation of data and its mapping to aesthetic attributes [58]. In the phyloseq package, functions having names beginning with “plot_” require a phyloseq object as input data, and return a ggplot2 graphics object. These plot_ functions support optional mapping of color, size, and shape aesthetics to sample or OTU variables — usually by providing the name of the variable or taxonomic rank as a character string (E.g. color = “SampleType”). Legends are automatically generated based on the data and aesthetic mappings (not true of the base R graphics), and all features of these graphics can be further modified in R via functions/options in the ggplot2 package.

**Figure 4:**
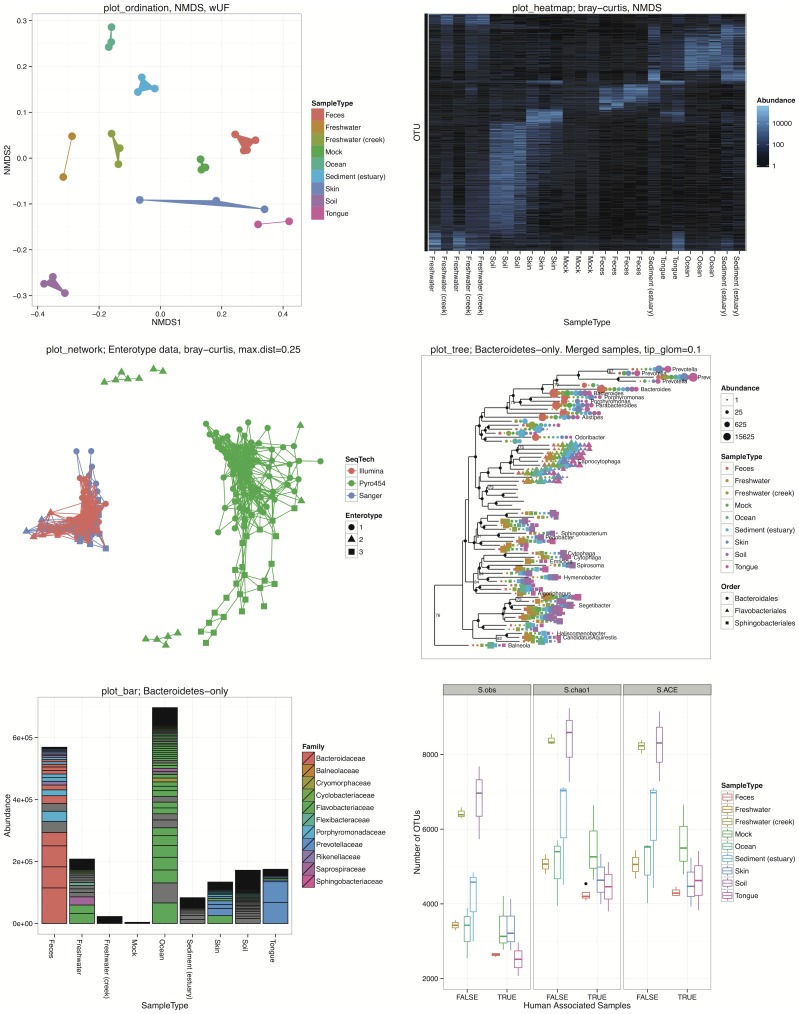
Graphic functions of the phyloseq package. The phyloseq class is an experiment-level data storage class defined by the phyloseq package for representing phylogenetic sequencing data. Most functions in the phyloseq package expect an instance of this class as their primary argument. See the phyloseq manual The Global Patterns [47] and Enterotypes [91] datasets are included with the phyloseq package. The Global Patterns data was preprocessed such that each sample was transformed to the same total read depth, and OTUs were trimmed that were not observed at least 3 times in 20% of samples or had a coefficient of variation ≤ 3.0 across all samples. For the plot_tree and plot_bar subplots, only the Bacteroidetes phylum is shown. Each subplot title indicates the plot function that produced it. Complete details for reproducing this figure are provided in File S2. All of these functions return a ggplot object that can be further customized/modified by tools in the ggplot2 package [57]. See additional descriptions of each function in the body text, and at the phyloseq homepage [39].

**Figure 5:**
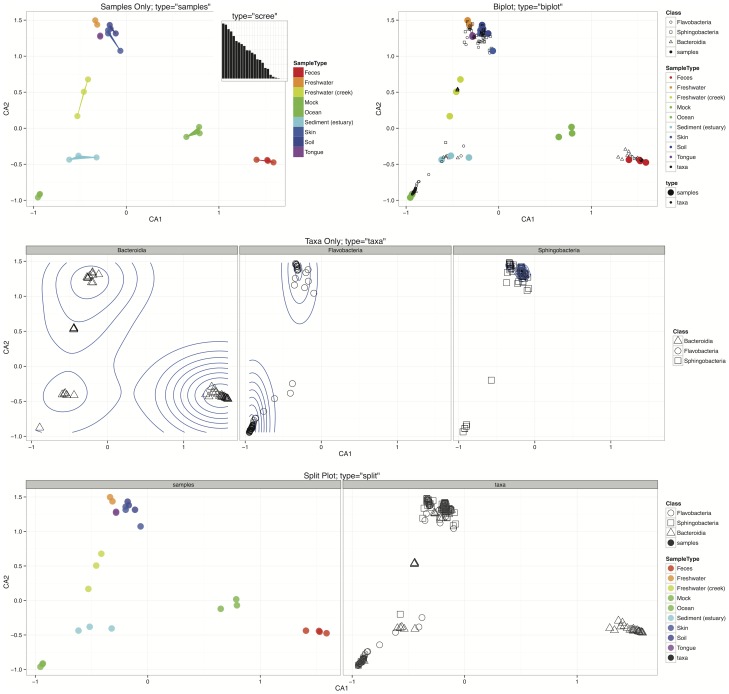
plot_ordination display methods included in phyloseq. Each panel uses a “Bacteroidetes-only” subset of the preprocessed “Global Patterns” dataset that was also used in Figure 4. The coordinates are derived from an unconstrained correspondence analysis [62]. Different panels illustrate different displays of the ordination results using the type argument to the plot_ordination function. (Top Left) Example of a samples-only display, with the “SampleType” mapped to the color aesthetic, and a filled-polygon layer to emphasize plot regions where sample types co-occur. (Top Left Insert) A “scree” plot of the eigenvalues associated with each axis, which indicates the proportion of total variability represented in each axis. (Top Right) Biplot representation in which samples and OTUs ordination results are overlaid. Clumps of OTUs appear to co-occur with different sample types, and some correlation with taxonomic phylum is also evident. (Middle) An OTUs-only plot that has been faceted (separated into panels) by class, with a two-dimensional density estimate overlain in blue. This view shows clearly a lack of association between the Sphingobacteria and Flavobacteria classes with fecal samples, which appear to be enriched in a subset of the Bacteroidia (relative to other OTUs in this Bacteroidetes-only dataset). Meanwhile, subsets of Bacteroidia appear to be enriched within multiple sample types. (Bottom) The “split” type for this graphic, in which both samples-only and OTUs-only plots are created, and shown side-by-side with one legend and shared vertical axis. Both the “biplot” and “split” options allow dual projections of both OTU- and sample-space.

The following list summarizes the key graphics-producing functions in phyloseq, which are also demonstrated in Figure 4, and in phyloseq's online tutorials [39]. File S2 provides the complete R code for creating Figures 4 and 5. We have also included some additional examples of graphics created by plot_ordination (Figure 5). They emphasize different aspects of ordination results, and the best choice depends heavily on characteristics of the data and research questions. The provided code also demonstrates a custom modification to the ggplot2 graphic, in this case the addition of a two-dimensional density estimate to the “OTUs-only” plot (File S2).

1. plot_ordination. This is the main function for plotting the results of an ordination. It currently supports four different representations of the ordination results: samples-only, OTUs-only, “biplot” (combined) representation, and “split”. A demonstration of these different options is provided in Figure 5. As can be seen in these examples, the “biplot” and “split” options support dual projections of both OTU- and sample-space. Additional parameters easily map the respective sample variable or taxonomic rank to color, size, or shape aesthetics.
2. plot_heatmap. This is a special implementation of the ordination-organized heat map similar to the NeatMap package [59]. Briefly, the abundance matrix is represented as a grid of colored tiles, with the color of the tiles mapped to the (usually transformed) abundance value. The ordering of the OTUs and sample indices in this representation is critical for discriminating any patterns. Traditionally, hierarchical clustering methods have been used for this organization; but, as Rajaram and Oono recently pointed out [59], this has the potential to misrepresent the data when deeply-branching elements are placed next to one another arbitrarily. Instead, the samples (and optionally, OTUs) are reordered based on their radial coordinate angle in the first two axes of an ordination. For the plot_heatmap function, any of the distances/ordinations supported by the distance and ordinate functions can be used, with the default being non-metric multidimensional scaling. Any arbitrary color scale can be selected, as well as any choice of numerical transformation for scaling the mapping of color shades to abundance.
3. plot_network. This function plots an igraph-class network [60] representing binary relationships between samples or OTUs. The network is calculated using the make_network function with phyloseq data as input and a desired ecological distance and threshold value. Unlike ordination, where most of the data structure is summarized by the relative position in two or more axes, the data is instead summarized by connections between samples (or OTUs) drawn with straight lines. Two samples are considered “connected” if the distance between them is less than a user-defined threshold. The relative position of points is optimized for the visual display of network properties, but is otherwise arbitrary. Any of the ecological distances supported by the distance function can be selected, and this can be a powerful representation of major clusters among samples or OTUs, provided the value of the distance threshold has been chosen carefully.
4. plot_tree. This function facilitates easy graphical rendering/investigation of the phylogenetic tree, with sample data overlaid. In some cases an annotated tree can be a powerful representation of an underlying evolutionary structure. The plot_tree function optionally places successive points next to the tips of the tree, indicating the samples in which each OTU was observed. These points can have their color, shape, and size aesthetics mapped to sample variables, revealing the correspondence of environmental variables on specific regions of the evolutionary tree. Standard ggplot2 customizations are supported, and this is, to our knowledge, the only function for ggplot2-based phylogenetic trees currently available in the CRAN/Bioconductor repositories. For phylogenetic sequencing of samples with large richness, some of the options in this function will be prohibitively slow to render or too dense to be interpretable, a drawback to summarizing phylogenetic sequencing data using trees. One suggestion is to either agglomerate or subset the data such that there are not more than 200 or so OTUs (tree tips) on a given plot, sometimes less depending on the complexity of the additional annotations being mapped to the tree. In many modern datasets 200 OTUs (or less) will be insufficient to summarize the entire dataset, in which case one or more of the other plot methods is suggested.
5. plot_bar. Although sometimes very complicated, a well-organized bar plot can be an effective graphical means for direct quantitative comparison of abundance values, and we note that statisticians generally discourage the use of pie-charts [61]. The plot_bar function takes as input a phyloseq dataset and a collection of arbitrary expressions for grouping the data based upon taxonomic rank and sample variables. The returned graphic represents each abundance value as the height of a rectangular block that is outlined by a thin black line and filled with the corresponding color of the user-specified sample or taxonomic variable, grey by default. Each of these OTU abundance rectangles corresponding to the same horizontal position (usually sample, or sample group) are stacked in order of abundance, such that the aggregate height of the stacked bar is also quantitatively informative.
6. plot_richness . This function creates plots of richness estimates of each sample in a phyloseq data object, allowing for horizontal grouping and color shading according to additional sample variables. Differences in richness (alpha diversity) between samples is often one of the first questions asked of phylogenetic sequencing data.

### Normalization and Standardization

In multivariate analyses such as PCA, large differences in variances between columns are corrected by standardizing each column; i.e. dividing each column by its standard deviation. Thus each column will have the same weight in the multivariate analysis. For OTU abundance tables, such a procedure is inappropriate as the disparities in column sums can be 100-fold. Methods based on chi-squared distances rather than variances deal with this by comparing weighted column profiles [62], computed as relative abundances for each OTU within a column, with the overall column sum retained as a weighting factor. However, chi-square distances are sums of squares and can be overly sensitive to outliers and sequencing “jackpot” effects such as those occurring in pyrosequencing data [63]. Bray-Curtis distances can be a useful alternative, as it is based on the 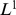 distance between profiles, as long as the differences in actual column sums are also accounted for in the final study. The other approach to the problem of disparities between column sums has been to subsample the over-abundant columns down to the same number as the smaller ones. However this results in a loss of information, rarely an optimal procedure in statistical contexts. This subsampling procedure is inspired by the popular idea of rarefaction in coverage studies first invented by Sanders [64], but has yet to be proved beneficial for all microbial community structures. The parallels between gene expression microarray analyses and microbial abundance analyses was mentioned in [65], which proposed several expression-inspired strategies for robustifying abundance measurements. The main points were that rankings and thresholding are important in the presence of noise and high variability in sequence depths. As in gene expression analysis filtering the OTUs is beneficial, especially in the latter multiple testing adjustments. The phyloseq package enables easy filtering and rank transformations in the same vein as robust multi-array averaging (rma) [66]. We provide further details in (McMurdie and Holmes, [67]).

### Confirmatory Analyses

Although useful for exploring and summarizing microbiome data, many of the graphics and ordination methods discussed here are not formal tests of any particular hypothesis. The most common framework for testing in microbiome studies is the comparisons of samples from different categories (e.g. healthy and obese; control and treated; different environments). Standard test statistics include the t-test, the paired permutation t-test, and ANOVA type tests based on F or pseudo-F statistics. However, microbiome data have two particularities. First, the raw abundance counts are never normally distributed, so the preferred methods are nonparametric. Second, there is contiguous information available about the relationships between OTUs, as well as for variables measured on the samples, so testing is sometimes more elaborate than a two-sample test. The hypergeometric test, also known as Fisher's exact test, is used in cases when we have a test statistic for each of the different OTUs. The goal is to confirm that a certain property of these significant OTUs is overrepresented compared to the general population of OTUs, often called “the universe”. For instance in Holmes et al [65] and Nelson et al [68] several phyla were shown to be significantly over-abundant in IBS rats as compared to healthy controls using this hypergeometric test.

An organizing principle in many nonparametric testing protocols is that the repetition of an analysis multiple times enables the user to control for multiple testing, or to evaluate the quality of estimators or the optimal values of tuning parameters. Modern confirmatory analyses currently depend on these repeated analyses under various data perturbation schemes, of which resampling, permutations, and Monte Carlo simulations are the most common. For instance the bootstrap uses many thousands of analyses of resampled data to address problems such as statistical stability or bias estimation [69], and can even provide confidence regions [69] for nonstandard parameters, such as phylogenetic trees [70]. Repeating analyses on permuted data can allow for control of the probability of encountering 1 or more false positives (falsely rejected nulls) among your group of simultaneous hypotheses, also called the Family Wise Error Rate (FWER). For instance, Westfall and Young's permutation-based **minP** procedure controls the FWER [71] and is implemented within the multtest package [72]. The phyloseq package interfaces with minP in multtest through a wrapper function, called mt. In the following example code we use the mt wrapper to control the FWER while simultaneously testing whether each OTU correlates with the “Enterotypes” classification of the samples. Note that we first remove samples that were not assigned an enterotype by the original authors (Table 1).
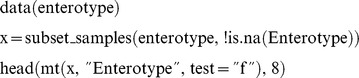



**Table 1:** Results from the mt function on the “Enterotypes” dataset.

genera	index	test stat	raw-p	adj-p
Prevotella	207	344.73	0.0001	0.0158
Bacteroides	203	85.01	0.0001	0.0158
Blautia	187	19.52	0.0001	0.0158
Bryantella	503	16.38	0.0001	0.0158
Parabacteroides	205	12.89	0.0001	0.0158
Alistipes	208	8.71	0.0002	0.0301
Bifidobacterium	240	9.29	0.0004	0.0560
Holdemania	201	7.64	0.0009	0.1146

The original “Enterotypes” dataset [91] (included in phyloseq) with OTU-wise testing of enterotype groups. Tests are a permutation-adjusted F-test using the Family-Wise Error Rate (FWER) as correction for multiple inferences (“adj-p” column). Not surprisingly, *Prevotella* and *Bacteroides* top the list, as they were major components of the “Enterotypes” classification described in the original article [91].

## Results and Discussion

As the complexity and sophistication of phylogenetic sequencing experiments continues to increase, it is clear that a “one-analysis fits all” approach is not sufficient. While it is often useful and convenient to have common analyses coupled within the application that decodes the sequences and clusters OTUs, we posit that a separate set of flexible open-source analytical tools is also needed that can be reproduced consistently by peers, and easily applied to new datasets and data sources. It should include a large library of statistical functions, and be independent of the choice of OTU-clustering method or sequencing technology. The phyloseq package helps satisfy this need by reducing the effort necessary to analyze OTU-clustered phylogenetic sequencing data via the R language and interactive computing environment.

### Reproducible Research and Sharing

In exploratory statistical work it is easy to produce biased results [73] through poorly chosen metrics or tests, a failure to properly control for multiple inferences, undisclosed data “pruning”, and probably many other means. Although not commonly required [74]–[76], an important defense against biased (or poorly-supported) findings is a higher standard for reproducibility in published research [77], in which journal articles are accompanied by sufficient data and software such that all presented analyses, tables, and figures can be reproduced exactly and with minimal effort [75]. In this context of highly-parallel phylogenetic-sequencing experiments, reproducible research can be partially facilitated by emerging standards for experimental design [78] and file format [37]. Virtual machine image and cloud-deployed “pipeline” analyses [11], [15], [19] can further increase accessibility of analyses by mitigating the need for expensive computing hardware while also avoiding complicated installation procedures. However, the use of publicly available “pipeline” tools does not fully meet the reproducibility standard unless accompanied with the complete code and data used in the analysis being published [75]. This is especially important when considering the many choices that are involved in decoding, OTU-clustering, and preprocessing; as well as the many varied approaches to incorporating sample covariates and performing multivariate analyses on complex data. The recent release of the HMP data and multiple articles on the results from their analyses underscore this fact. Thresholding and noise filtering were done independently by each team, but no overall robustness study was performed [79]. Changes early in the analysis pipeline could have downstream effects that are now prohibitively difficult or impossible to evaluate. Generally speaking, the preprocessing of OTU abundance data through filtering, normalizing, centering, shrinking, and other transformations is a common practice and necessary for analysis [66], but varies widely among researchers and is often difficult to reproduce. This is particularly true when the preprocessing transformations are the result of “manual” adjustments in a spreadsheet, custom code/script that is not included in the publication, or random subsampling (“rarefying” to even sequencing effort) with no reported seed. A related example is the (often not-so) reproducible choice of tuning parameters and perturbation-based statistical validation procedures, allowing for the easy testing of alternatives and robustness of results. To a large extent this revisits many of the same issues of reproducible research [80]–[82] that have been addressed over the last decade for the analysis of microarray data [66], and for which there are many proven tools already available in Bioconductor/R. The emphasis of preprocessing tools in phyloseq is intended to decrease the extent to which these steps constitute opaque and idiosyncratic efforts by investigators, while making the results of different studies more comparable.

One of the goals of the phyloseq project is to help close the gap in reproducible research that presently exists between pipeline results and the additional analyses required by investigators. This can be achieved when phyloseq is used (possibly with other R packages) in conjunction with documentation tools such as Sweave [32], knitr [33], iPython [83] Notebook invoking the rmagic extension, or “ R flavored markdown” (RFM) [84]. The Sweave-format approach is part of the reproducible research standards strongly encouraged by the journal *Biostatistics* [81], as well as many disciplines related to statistics and bioinformatics [77], [85]. The recently-described RFM format and iPython Notebook can also work very well for cases where a web-browser is a satisfactory documentation delivery medium, with RFM being our preferred source format for publishing reproducible online tutorials with embedded code and figures (HTML5) [39], [86]. We emphasize that the benefits of reproducibility are not contingent on “pretty” code [87], and we encourage researchers in the field to make their code available even if they feel insecure about its programmatic elegance. As an illustrative example, we have made available the Sweave (.Rnw) and supporting files required to completely reproduce this article, including especially the complete source as an RFM file (.Rmd) with its associated output HTML file, both of which provide the preprocessing steps and graphics commands needed to exactly reproduce each figure (File S2). We have also published a GitHub repository dedicated to reproducible demonstrations of analyses with phyloseq [86].

### Extending phyloseq

It is important to note that the new phyloseq-class is a significant departure from the originally-proposed phyloseq-class structure [31], which used nested multiple inheritance and a naming convention. It was a valid approach in principle, but was an overly complex approach for the goal of representing a phylogenetic sequencing experiment as a single object. The updated phyloseq-class is simple to extend for developers and easy to explain to users (Figure 3). In general, the downstream analysis and plotting functions that might operate on an instance of the phyloseq-class do not need to (re)perform common validity checks because these checks are consolidated as part of the phyloseq-constructor method.

Analysis tools available in R but not explicitly wrapped in phyloseq are nevertheless available to users and developers via accessors and other data infrastructure tools. This leverages the fact that phyloseq data components are based on standard R data classes and easily used in other package settings in R. For example, we have included example code that illustrates the use of the bioenv function from the vegan package, starting with data represented by the phyloseq-class (See File S2 for code, and the phyloseq demo [86]). Similarly, as an open-source package in an open language/framework (R), phyloseq can be easily included at the relevant steps in pipelines, workbenches, and GUIs now under active development (E.g. ClovR [15], MG-RAST [19], QIIME [11], mcaGUI [88]). This represents a means for investigators with limited programming literacy to still benefit from some of the tools included in, or facilitated by, phyloseq.

## Conclusions

The phyloseq project is a new open-source software tool for statistical analysis of phylogenetic sequencing data within the R programming language and environment. The tools in phyloseq make it easy to read the data output of several of the most common OTU clustering pipelines, and also represents this data in a unified, integrated form amenable to many modern analysis methods. With this integrated representation of the data it is easy to use supervised methods — such as canonical correspondence analysis, discriminant correspondence analysis, sparse linear discriminant analysis, etc. — to explain clinical or environmental response variables. We hope that this will provide a gateway for users to take their analyses towards more robust nonparametric alternatives to classical least squares methods, and allow them to interact graphically with their data more easily and efficiently. By leveraging existing R infrastructure for reproducible research, the phyloseq project also enables reproducible preprocessing, analysis, and publication-quality graphics production — such that it is easy to document, share, and modify analyses of phylogenetic sequencing data. The phyloseq package is released on Bioconductor [34] and developed collaboratively on GitHub [39].

## Availability and Requirements

**Project name:** phyloseq

**Project Stable Release:** http://www.bioconductor.org/packages/release/bioc/html/phyloseq.html

**Project Home Page:** http://joey711.github.com/phyloseq/

**Project Issue Tracker:** https://github.com/joey711/phyloseq/issues

**Project Demo Page:** http://joey711.github.com/phyloseq-demo/

**Operating System(s):** Platform Independent

**Programming Language(s):** R

**Other Requirements:** R, R packages (ade4, ape, Biostrings, foreach, ggplot2, igraph0, multtest, picante, plyr, reshape, RJSONIO, scales, vegan)

**License:** AGPL-3

## Supporting Information

File S1 **Summary of comparison between phyloseq and currently available software.** This PDF file contains a table summarizing a comparison of supported capabilities between phyloseq and QIIME [11], mothur [12], and the pair of packages OTUbase [35] and mcaGUI [88]. A “+” or “–” indicates that the capability is not directly supported, respectively. A symbol or word instead of “+” implies that the capability is supported, but with an extra caveat or detail, further defined below the table, if necessary. This is not a comprehensive summary of the capabilities of each packages, but rather the capabilities of relevance to this article. The abbreviations CA, DCA, RDA, and DPCoA stand for the ordination methods correspondence analysis, detrended correspondence analysis, redundancy analysis, and double principal coordinates analysis, respectively. Note that in some cases the capabilities deemed “+” in this table are only supported for amplicon sequencing based data, sometimes from a specific sequencing platform and with the 16S rRNA gene as target. However, the phyloseq package is implemented at a stage in the analysis process that can be more generally applied to any phylogenetic sequencing, including non-standard amplicon targets, shotgun metagenome sequencing, etc.
(PDF)

File S2 **Source materials for reproducing this manuscript.** This is a compressed .zip directory containing the main source file in Sweave .Rnw format [32], as well as the additional files necessary to completely recreate the original manuscript submitted to PLoS ONE. For the uninitiated, Sweave is a R/LaTeX2e interleaved hybrid language format [32] that allows advanced typesetting description to accompany R code and its output (including graphics). Also included is the RFM source file that was used to create Figures 4 and 5, and its accompanying HTML output that includes additional documentation details, links, and intermediate graphics. This latter file is “sourced” (re-run) by the Sweave commands if any of the expected output files are missing. This supporting information zip file also includes R code (at the end of the RFM/HTML files) that demonstrates how to use a phyloseq data object as an argument to other R functions. In this particular example, the bioenv function from the vegan package [92] is demonstrated.
(ZIP)
