# Supplementary material for: phyloseq: An R Package for Reproducible Interactive Analysis and Graphics of Microbiome Census Data
Source: PLoS One. 2013 Apr 22;8(4):e61217. doi: 10.1371/journal.pone.0061217 (PMC3632530; doi:10.1371/journal.pone.0061217)
Supplement: File S1 — Summary of comparison between phyloseq and currently available software. This PDF file contains a table summarizing a comparison of supported capabilities between phyloseq and QIIME [11], mothur [12], and the pair of packages OTUbase [35] and mcaGUI [88]. A “+” or “–” indicates that the capability is not directly supported, respectively. A symbol or word instead of “+” implies that the capability is supported, but with an extra caveat or detail, further defined below the table, if necessary. This is not a comprehensive summary of the capabilities of each packages, but rather the capabilities of relevance to this article. The abbreviations CA, DCA, RDA, and DPCoA stand for the ordination methods correspondence analysis, detrended correspondence analysis, redundancy analysis, and double principal coordinates analysis, respectively. Note that in some cases the capabilities deemed “+” in this table are only supported for amplicon sequencing based data, sometimes from a specific sequencing platform and with the 16S rRNA gene as target. However, the phyloseq package is implemented at a stage in the analysis process that can be more generally applied to any phylogenetic sequencing, including non-standard amplicon targets, shotgun metagenome sequencing, etc. (PDF) [file pone.0061217.s001.pdf]

Category	Capability	phyloseq	QIIME	mothur	OTUbase/mcaGUI
Sequence Processing	decode barcoded reads	-	+	+	-
	cluster sequence reads into OTUs	-	+	+	-
	chimera filtering	-	+	+	-
Phylogenetics	reference alignment of OTU reps	-	+	+	-
	taxonomic classification	-	+	+	-
	store/manipulate phylogenetic trees	+	+	+	-
Ecology	multiple community distance/similarity metrics	+	+	+	+
	parallel Weighted and Unweighted UniFrac	+	+	+	-
Ordination	principal coordinates analysis (MDS/PCoA)	+	+	+	+
	nonmetric multidimensional scaling (NMDS)	+	+	+	-
	principal components analysis (PCA)	+	-	+	-
	CA, DCA, RDA, DPCoA	+	-	-	-
Graphics	ordination plot	+	P	+	P
	heat map	+	+	+	-
	quantitative abundance bar/pie plot	+	+	-	+
	multi-sample richness estimates plot	+	+	-	+
	annotated tree plot	+	-	+	-
	network plot	+	-	-	-
	integration with object oriented graphics	+	-	-	-
Validation	multiple inference correction	+	-	-	-
Reproducibility	tools for collaboration/reproduction/sharing	R	iP	S	R
Computation	language interface	R	python	C++	R
Documentation	web-based tutorials/wiki	+	+	+	-

P – PCoA-only
R – R Flavored Markdown, Sweave, etc.
iP – iPython Notebook, etc.
S – shell scripts
