# Supplementary material for: phyloseq: An R Package for Reproducible Interactive Analysis and Graphics of Microbiome Census Data
Source: PLoS One. 2013 Apr 22;8(4):e61217. doi: 10.1371/journal.pone.0061217 (PMC3632530; doi:10.1371/journal.pone.0061217)
Supplement: File S2 — Source materials for reproducing this manuscript. This is a compressed .zip directory containing the main source file in Sweave .Rnw format [32], as well as the additional files necessary to completely recreate the original manuscript submitted to PLoS ONE. For the uninitiated, Sweave is a R/LaTeX2e interleaved hybrid language format [32] that allows advanced typesetting description to accompany R code and its output (including graphics). Also included is the RFM source file that was used to create Figures 4 and 5, and its accompanying HTML output that includes additional documentation details, links, and intermediate graphics. This latter file is “sourced” (re-run) by the Sweave commands if any of the expected output files are missing. This supporting information zip file also includes R code (at the end of the RFM/HTML files) that demonstrates how to use a phyloseq data object as an argument to other R functions. In this particular example, the bioenv function from the vegan package [92] is demonstrated. (ZIP) [file pone.0061217.s002.zip › phyloseq-article-source-files-figs-code-03/phyloseq_plos1_2012-source-doc.html]

Complete Source Code for 2013 phyloseq Manuscript


# Complete Source Code for 2013 phyloseq Manuscript

This is an R Markdown document creating the graphics in this manuscript. Markdown is a simple formatting syntax for authoring web pages. Try RStudio for writing and interpreting R Markdown documents.

# Load required packages

```
library("phyloseq")
library("ggplot2")
library("scales")
library("grid")
```

Should be using phyloseq version `1.3.20` or greater for reliable behavior. See
the phyloseq homepage

```
packageVersion("phyloseq")
```

```
## [1] '1.3.20'
```

```
packageVersion("ggplot2")
```

```
## [1] '0.9.3'
```

```
packageVersion("scales")
```

```
## [1] '0.2.3'
```

```
packageVersion("grid")
```

```
## [1] '2.15.2'
```

```
packageVersion("knitr")
```

```
## [1] '1.1'
```

# Preprocess phyloseq data for plotting

The following is the complete set of preprocessing steps that was applied to the `GlobalPatterns` OTU counts prior to creating the figures in the 2013 phyloseq manuscript. See the tutorial on preprocessing for further details and examples.

Load the `GlobalPatterns` dataset into the workspace.

```
data("GlobalPatterns")
```

Define a human versus non-human categorical variable, store this as a 2-category factor in the sample data.

```
sample_data(GlobalPatterns)$human = factor(sample_data(GlobalPatterns)$SampleType %in% 
    c("Feces", "Mock", "Skin", "Tongue"))
```

Remove taxa not seen more than 3 times in at least 20% of the samples. This helps protect against an OTU with small mean & trivially large Coefficient of Variation. Save this as new phyloseq data object, `GP`.

```
GP = filter_taxa(GlobalPatterns, function(x) sum(x > 3) > (0.2 * length(x)), 
    TRUE)
```

Transform abundances to the median sequencing depth.

```
total = median(sample_sums(GP))
standf = function(x, t = total) round(t * (x/sum(x)))
gps = transform_sample_counts(GP, standf)
```

Filter the taxa using a cutoff of `3.0` for the Coefficient of Variation.

```
gpsf = filter_taxa(gps, function(x) sd(x)/mean(x) > 3, TRUE)
```

Subset the data to “Bacteroidetes-only”“, used in some plots, called `gpsfb`.

```
gpsfb = subset_taxa(gpsf, Phylum == "Bacteroidetes")
```

Save the preprocessed data, in case you want to skip this step in the future.

```
save(gpsf, gpsfb, file = "gp-ex.RData")
```

Check for existence of submit-main directory. If not present, create it.

```
main_figure_dir_name = "submit-main"
if (!file.exists(main_figure_dir_name)) {
    dir.create(main_figure_dir_name)
}
```

---

# Figure 4

---

# Create graphics for main plot

Create the six example plots in the main plot figure for phyloseq manuscript

Define the base theming you want to use across all plots

```
theme_set(theme_bw())
```

## 1 plot\_ordination

See the plot\_ordination tutorial for more details, examples.

Perform NMDS on weighted UniFrac distance. This is a Bacteroidetes-only subset of the data.

```
gpsfb.wUF = distance(gpsfb, "unifrac", weighted = TRUE)
gpsfb.NMDS = ordinate(gpsfb, "NMDS", gpsfb.wUF)
```

```
## Run 0 stress 0.08582 
## Run 1 stress 0.1374 
## Run 2 stress 0.1374 
## Run 3 stress 0.08872 
## Run 4 stress 0.08582 
## ... New best solution
## ... procrustes: rmse 0.002305  max resid 0.008574 
## *** Solution reached
```

```
p1 = plot_ordination(gpsfb, gpsfb.NMDS, "samples", color = "SampleType", title = "plot_ordination, NMDS, wUF")
p1
```

Add fill to emphasize regions and overlap

```
p1 = p1 + geom_polygon(aes(fill = SampleType)) + geom_point(size = 5)
p1
```

## 2 plot\_heatmap

See the plot\_heatmap tutorial for more details, examples.

Create the heatmap on the full dataset, using Bray-Curtis distance and NMDS.

```
title = "plot_heatmap; bray-curtis, NMDS"
p2 = plot_heatmap(gpsf, "NMDS", "bray", "SampleType", trans = log_trans(10), 
    title = title)
p2
```

```
p2 = p2 + theme(axis.text.x = element_text(size = 10, angle = -90, hjust = 0))
p2
```

## 3 plot\_network

Example with the `plot_network` function using an example from the `enterotype` dataset. See the plot\_network tutorial for further details and examples.

Load the `enterotype` dataset for this one, and clean it of the samples for which the original authors did not provide an "Enterotypes” designation.

```
data("enterotype")
enterotype = subset_samples(enterotype, !is.na(Enterotype))
```

Now create the `igraph` network object, and build the network graphic.

```
mxdist = 0.25
ig = make_network(enterotype, "samples", "bray", max.dist = mxdist)
title = paste("plot_network; Enterotype data, bray-curtis, max.dist=", mxdist, 
    sep = "")
p3 = plot_network(ig, enterotype, "samples", color = "SeqTech", shape = "Enterotype", 
    line_weight = 0.5, label = NULL, title = title, line_alpha = 1)
p3
```

## 4 plot\_tree

Example using `plot_tree` with the Bacteroidetes-only data, `gpsfb`, which will be further consolidated/simplified. See the plot\_tree tutorial for more details, examples.

Define the title for your tree.

```
title = "plot_tree; Bacteroidetes-only. Merged samples, tip_glom=0.1"
```

Coerce the node labels to be proper bootstraps by rm perplexing suffix.

```
head(phy_tree(gpsfb)$node.label)
```

```
## [1] "0.768.276" "0.361.73"  "0.924.733" "0.228.73"  "0.982.388" "0.957.400"
```

```
phy_tree(gpsfb)$node.label = substr(phy_tree(gpsfb)$node.label, 1, 4)
head(phy_tree(gpsfb)$node.label)
```

```
## [1] "0.76" "0.36" "0.92" "0.22" "0.98" "0.95"
```

Not finished for the figure, and too busy/crowded a graphic, but let's document what that looks like, anyway.

```
plot_tree(gpsfb, "sampledodge", nodeplotboot(), "Order", "human", "abundance", 
    title = title, ladderize = "left")
```

Yep, too many tips for display in the available space in the main plot of the manuscript. Consolidate the data for the tree into new object, called `tggpsfb`. First by agglomerating OTUs that are similar enough to have a patristic distance less than `0.1`. Second, merge samples that come from the same `SampleType` (environment), as we are mostly interested in comparing between environments, and we already know that the microbiome profiles from the same `SampleType` in this dataset are very similar relative to the between `SampleType` differences.

```
tggpsfb = tip_glom(gpsfb, NULL, 0.1)
tggpsfb = merge_samples(tggpsfb, "SampleType")
```

Repair the data factors that we want to use. They tend to be coerced to something other than factors during `data.frame` `merge`.

```
sample_data(tggpsfb)$human = factor(sample_names(tggpsfb) %in% c("Feces", "Mock", 
    "Skin", "Tongue"))
sample_data(tggpsfb)$SampleType = factor(sample_names(tggpsfb))
```

Compare two different aesthetic mappings for the tree. We will go with the latter for the publication graphic, but it's interesting to compare the two.

```
plot_tree(tggpsfb, "sampledodge", nodeplotboot(85L, 60L), "Order", "human", 
    "abundance", title = title, ladderize = "left")
```

```
p4 = plot_tree(tggpsfb, "sampledodge", nodeplotboot(85L, 60L), "SampleType", 
    "Order", "abundance", title = title, ladderize = "left", label.tips = "Genus")
p4
```

## 5 plot\_bar

Example of `plot_bar` using the Bacteroidetes-only subset, `gpsfb`. The horizontal position is mapped to `SampleType`, the vertical axis mapped to OTU abundance value, and the box fill is mapped to taxonomic family of each OTU. See the plot\_bar tutorial for more details, examples.

```
title = "plot_bar; Bacteroidetes-only"
p5 = plot_bar(gpsfb, "SampleType", "Abundance", "Family", title = title)
p5
```

## 6 plot\_richness

Example of `plot_richness` using the original unfiltered `GlobalPatterns` dataset. See the plot\_richness tutorial for more details, examples.

```
plot_richness(GlobalPatterns, "human", "SampleType", title = "plot_richness", 
    shsi = TRUE)
```

```
p6 = plot_richness(GlobalPatterns, "human", "SampleType", title = "plot_richness")
p6
```

```
p6 + geom_boxplot(data = p6$data, aes(x = human, y = value, color = NULL), alpha = 0.1)
```

```
p6 = ggplot(p6$data, aes(x = human, y = value, color = SampleType)) + geom_boxplot(alpha = 0.1)
p6
```

```
p6 = p6 + facet_wrap(~variable, nrow = 1) + xlab("Human Associated Samples") + 
    ylab("Number of OTUs")
p6
```

# Combine main plot and save

This requires the grid package, which was loaded at the very beginning on this tutorial. The following code combines each of the previous individual plots (the final versions) into one larger graphic for publication, especially `grid.newpage`, `pushViewport`, and `viewport`.
The first step is to create the viewport defining the graphics grid. Then loop through and add each of the previous ggplot graphics to this PDF/viewport grid.

First embed an example of the graphic directly into this tutorial web document.

```
grid.newpage()
pushViewport(viewport(layout = grid.layout(3, 2)))
i = 1
for (x in 1:3) {
    for (y in 1:2) {
        p_i = eval(parse(text = paste("p", i, sep = "")))
        print(p_i, vp = viewport(layout.pos.row = x, layout.pos.col = y))
        i = i + 1
    }
}
```

```
# Always turn-off/close-connection-with the graphics device when finished.
dev.off()
```

```
## null device 
##           1
```

Now repeat the same code as above, but brace with the `pdf` and `dev.off` functions in order to send the combined graphic to a file for publication. Note that `png()` could also work in place of `pdf()` if raster graphic needed.

```
pdf(paste(main_figure_dir_name, "phyloseq-plot-main.pdf", sep = "/"), width = 16, 
    height = 20)
grid.newpage()
pushViewport(viewport(layout = grid.layout(3, 2)))
i = 1
for (x in 1:3) {
    for (y in 1:2) {
        p_i = eval(parse(text = paste("p", i, sep = "")))
        print(p_i, vp = viewport(layout.pos.row = x, layout.pos.col = y))
        i = i + 1
    }
}
# Always turn-off/close-connection-with the graphics device when finished.
dev.off()
```

```
## pdf 
##   2
```

---

# Figure 5

---

# Ordination plots

Make ordination plots for ordination figure.

Perform correspondence analysis.

```
GP.ca = ordinate(gpsfb, method = "CCA")
```

Define the default alpha (transparency, `{0, 1}`) to use for plot points

```
alpha = 0.75
```

Define a default color pallete using `rainbow()`. In all plots, color is mapped to `SampleType` variable, sometimes plus an extra for taxa or samples.

```
color_var = get_variable(gpsfb, "SampleType")
color_pal = rainbow(length(levels(color_var)) + 1)
names(color_pal) = c(levels(color_var), "taxa")
color_pal["taxa"] = "black"
```

Similarly, define a standard shape-scale

```
shape_var = get_taxa_unique(gpsfb, "Class")
shape_scale = c(0:2, 19)
names(shape_scale) = c(shape_var, "samples")
```

## 0 plot\_ordination - scree plot

Must have loaded the grid package for the `unit` function (we did at the beginning).

```
p0 = plot_ordination(gpsfb, GP.ca, "scree", title="type=\"scree\"")
p0
```

Modify some aspects of the theme for this plot, especially removing certain elements from the axes. Unit arguments for `plot.margin` have the form `c("top", "right", "bottom", "left")`.

```
b = element_blank()
p0 = p0 + theme(axis.title = b, axis.ticks = b, axis.text = b, panel.background = b, 
    plot.margin = unit(c(0, 0, -1, 0), "lines"))
p0
```

## 1 plot\_ordination - samples-only

```
p1 = plot_ordination(gpsfb, GP.ca, "samples", color="SampleType", title="Samples Only; type=\"samples\"") 
p1
```

Add the enlarged point size

```
p1 = p1 + geom_point(size = 5, alpha = alpha)
p1
```

Remove the original non-transparent, small-size `geom_point` layer.

```
p1$layers = p1$layers[-1]
p1
```

Add the filled polygon

```
p1 = p1 + geom_polygon(aes(fill = SampleType))
p1
```

Set the manual color scale from `color_pal`.

```
p1 = p1 + scale_colour_manual(values = color_pal) + scale_fill_manual(values = color_pal)
p1
```

## 2 plot\_ordination - biplot

```
p2 = plot_ordination(gpsfb, GP.ca, "biplot", color="SampleType", shape="Class", title="Biplot; type=\"biplot\"") 
p2
```

Re-order the color elements in legend.

```
stleg = as(p2$data$SampleType, "character")
stleg = factor(stleg, levels = c(levels(get_variable(gpsfb, "SampleType")), 
    "taxa"))
p2$data$SampleType = stleg
p2
```

Add the layer defining the custom color scale/pallete

```
p2 = p2 + scale_colour_manual(values = color_pal)
```

```
## Scale for 'colour' is already present. Adding another scale for 'colour',
## which will replace the existing scale.
```

```
p2
```

Re-order the shape elements in legend.

```
cleg = as(p2$data$Class, "character")
cleg = factor(cleg, levels = c(unique(cleg[cleg != "samples"]), "samples"))
p2$data$Class = cleg
p2
```

Set manual shape scale, using previously-defined `shape_var`.

```
p2 = p2 + scale_shape_manual(values = shape_scale)
p2
```

## 3 plot\_ordination - taxa only

```
p3 = plot_ordination(gpsfb, GP.ca, "taxa", shape="Class", title="Taxa Only; type=\"taxa\"")
p3
```

Separate the data into panels by taxonomic class of each OTU by adding a facet layer using the `facet_wrap` function.

```
p3 = p3 + facet_wrap(~Class, nrow = 1)
p3
```

Add a layer with larger-sized points using `geom_point`. It will use the default aesthetic mapping and data when invoked like this.

```
p3 = p3 + geom_point(size = 5)
p3
```

Remove the original point layer that had small-sized points.

```
p3$layers = p3$layers[-1]
p3
```

Manually define the shape scale that you want to use (as in, which shapes, and which order).

```
p3 = p3 + scale_shape_manual(values = shape_scale)
p3
```

Add the 2-D density estimation.

```
p3 = p3 + geom_density2d()
p3
```

## 4 plot\_ordination - split plot

```
p4 = plot_ordination(gpsfb, GP.ca, "split", color="SampleType", shape="Class", title="Split Plot; type=\"split\"")
p4
```

Adjust point size and color scale

```
p4 = p4 + geom_point(size = 5, alpha = alpha)
p4
```

Remove the original smaller-point-size layer

```
p4$layers = p4$layers[-1]
p4
```

Set color scales to manual, `color_pal`

```
p4 = p4 + scale_colour_manual(values = color_pal) + scale_fill_manual(values = color_pal)
p4
```

Re-order the color elements in legend.

```
stleg = as(p4$data$SampleType, "character")
stleg = factor(stleg, levels = c(levels(get_variable(gpsfb, "SampleType")), 
    "taxa"))
p4$data$SampleType = stleg
p4
```

Re-order the shape elements in legend.

```
cleg = as(p4$data$Class, "character")
cleg = factor(cleg, levels = c(unique(cleg[cleg != "samples"]), "samples"))
p4$data$Class = cleg
p4
```

Adjust the shape values

```
p4 = p4 + scale_shape_manual(values = shape_scale)
p4
```

## Create combined ordination graphic

Requires the grid package. First test it out and embed in this tutorial page.

```
grid.newpage()
pushViewport(viewport(layout = grid.layout(3, 2)))
print(p1, vp = viewport(layout.pos.row = 1, layout.pos.col = 1))
print(p2, vp = viewport(layout.pos.row = 1, layout.pos.col = 2))
print(p3, vp = viewport(layout.pos.row = 2, layout.pos.col = 1:2))
print(p4, vp = viewport(layout.pos.row = 3, layout.pos.col = 1:2))
# Add the scree plot to the first ordination plot, `p1`
subvp <- viewport(width = 0.1, height = 0.1, x = 0.34, y = 0.925)
print(p0, vp = subvp)
```

```
# Close the device.
dev.off()
```

```
## null device 
##           1
```

Now send the same graphics to a PDF file for publication, by bracing the previous graphics commands with `pdf()` and `dev.off()` commands.

```
pdf(paste(main_figure_dir_name, "phyloseq-plot-ordination.pdf", sep = "/"), 
    width = 18, height = 17)
grid.newpage()
pushViewport(viewport(layout = grid.layout(3, 2)))
print(p1, vp = viewport(layout.pos.row = 1, layout.pos.col = 1))
print(p2, vp = viewport(layout.pos.row = 1, layout.pos.col = 2))
print(p3, vp = viewport(layout.pos.row = 2, layout.pos.col = 1:2))
print(p4, vp = viewport(layout.pos.row = 3, layout.pos.col = 1:2))
# Add the scree plot to the first ordination plot, `p1`
subvp <- viewport(width = 0.1, height = 0.1, x = 0.34, y = 0.925)
print(p0, vp = subvp)
# Close the device.
dev.off()
```

```
## pdf 
##   2
```

---


---

# Using phyloseq data in other packages

Example porting data represented by the phyloseq-class for use in other packages, in this case the vegan package.

```
library("vegan")
packageVersion("vegan")
```

```
## [1] '2.0.6'
```

Define a function for extracting the OTU table from a phyloseq object and coercing it into a matrix class oriented in a way that vegan functions expect.

```
veganotu <- function(physeq) {
    OTU <- otu_table(physeq)
    if (taxa_are_rows(OTU)) {
        OTU <- t(OTU)
    }
    return(as(OTU, "matrix"))
}
```

Now reload the `enterotype` dataset.

```
data("enterotype")
```

Need to identify continuous variables

```
keepvariables = which(sapply(sample_data(enterotype), is.numeric))
print(keepvariables)
```

```
## Age 
##   8
```

```
ent <- data.frame(sample_data(enterotype))[keepvariables]
```

Well, that's only one continuous variable, so let's add some simulated continuous variables to make this slightly more interesting.

```
sim1 = sample(seq(0, 1, 0.02), nsamples(enterotype), TRUE)
sim2 = sample(seq(0, 1, 0.02), nsamples(enterotype), TRUE)
```

Add these simulated variables to sample-data

```
ent$sim1 = sim1
ent$sim2 = sim2
```

Since there were ages missing, let's simulate those missing ages, too

```
ent$Age[is.na(ent$Age)] = sample(1:92, sum(is.na(ent$Age)), TRUE)
```

Now try the vegan function, `bioenv`

```
bioenv(veganotu(enterotype), ent)
```

```
## 
## Call:
## bioenv(comm = veganotu(enterotype), env = ent) 
## 
## Subset of environmental variables with best correlation to community data.
## 
## Correlations:      spearman 
## Dissimilarities:   bray 
## 
## Best model has 2 parameters (max. 3 allowed):
## Age sim1
## with correlation  0.01975
```
