# Supplementary figures and images for: phyloseq: An R Package for Reproducible Interactive Analysis and Graphics of Microbiome Census Data
Source: PLoS One. 2013 Apr 22;8(4):e61217. doi: 10.1371/journal.pone.0061217 (PMC3632530; doi:10.1371/journal.pone.0061217)

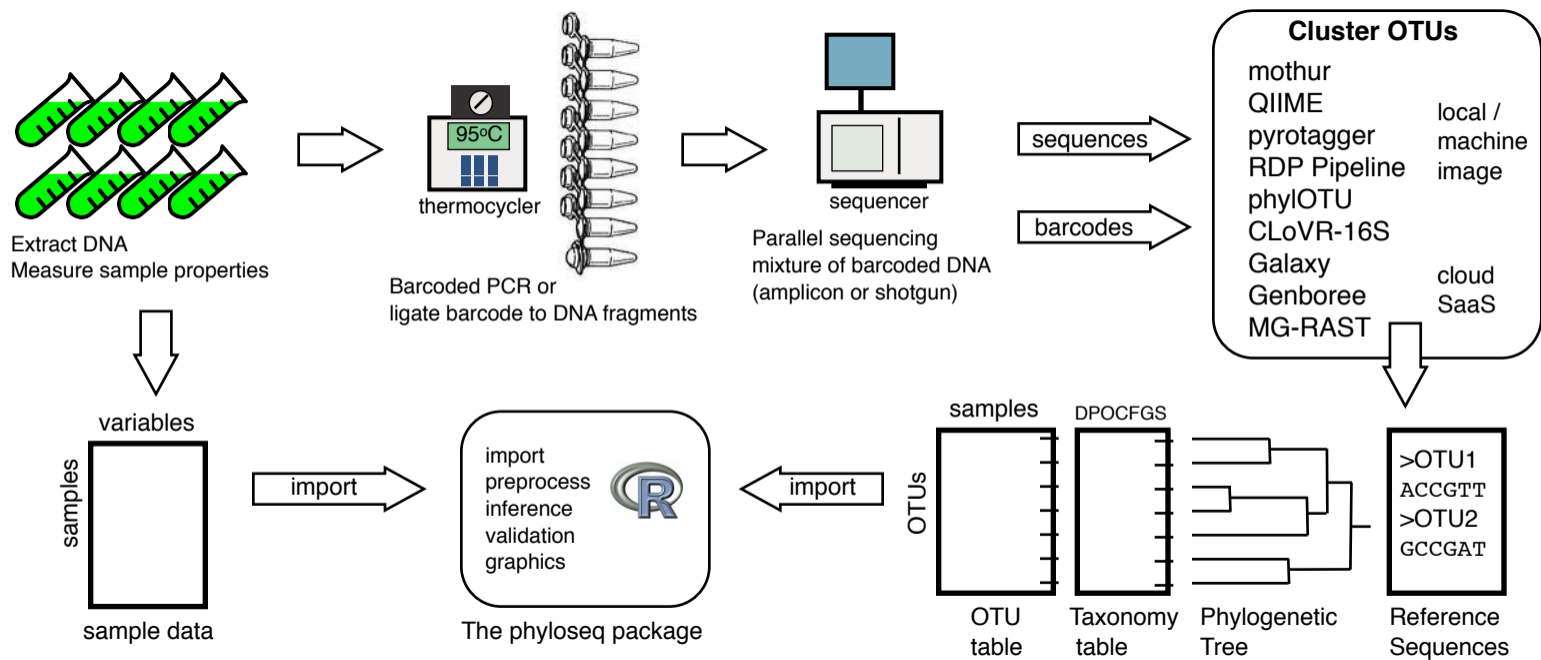

Supplement: File S2 — Source materials for reproducing this manuscript. This is a compressed .zip directory containing the main source file in Sweave .Rnw format [32], as well as the additional files necessary to completely recreate the original manuscript submitted to PLoS ONE. For the uninitiated, Sweave is a R/LaTeX2e interleaved hybrid language format [32] that allows advanced typesetting description to accompany R code and its output (including graphics). Also included is the RFM source file that was used to create Figures 4 and 5, and its accompanying HTML output that includes additional documentation details, links, and intermediate graphics. This latter file is “sourced” (re-run) by the Sweave commands if any of the expected output files are missing. This supporting information zip file also includes R code (at the end of the RFM/HTML files) that demonstrates how to use a phyloseq data object as an argument to other R functions. In this particular example, the bioenv function from the vegan package [92] is demonstrated. (ZIP) [file pone.0061217.s002.zip › phyloseq-article-source-files-figs-code-03/experimental-physeq-overview-graphic.pdf]

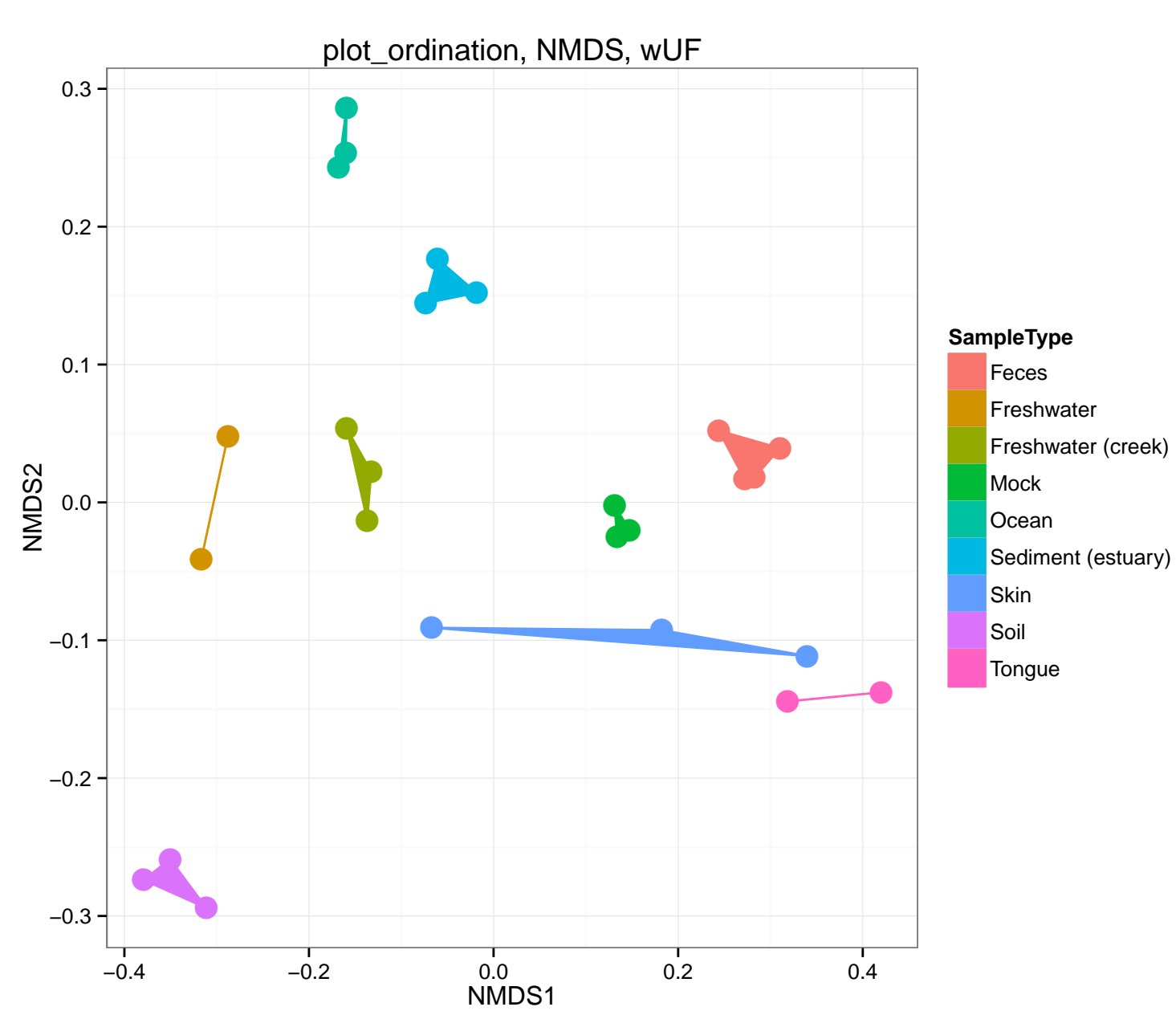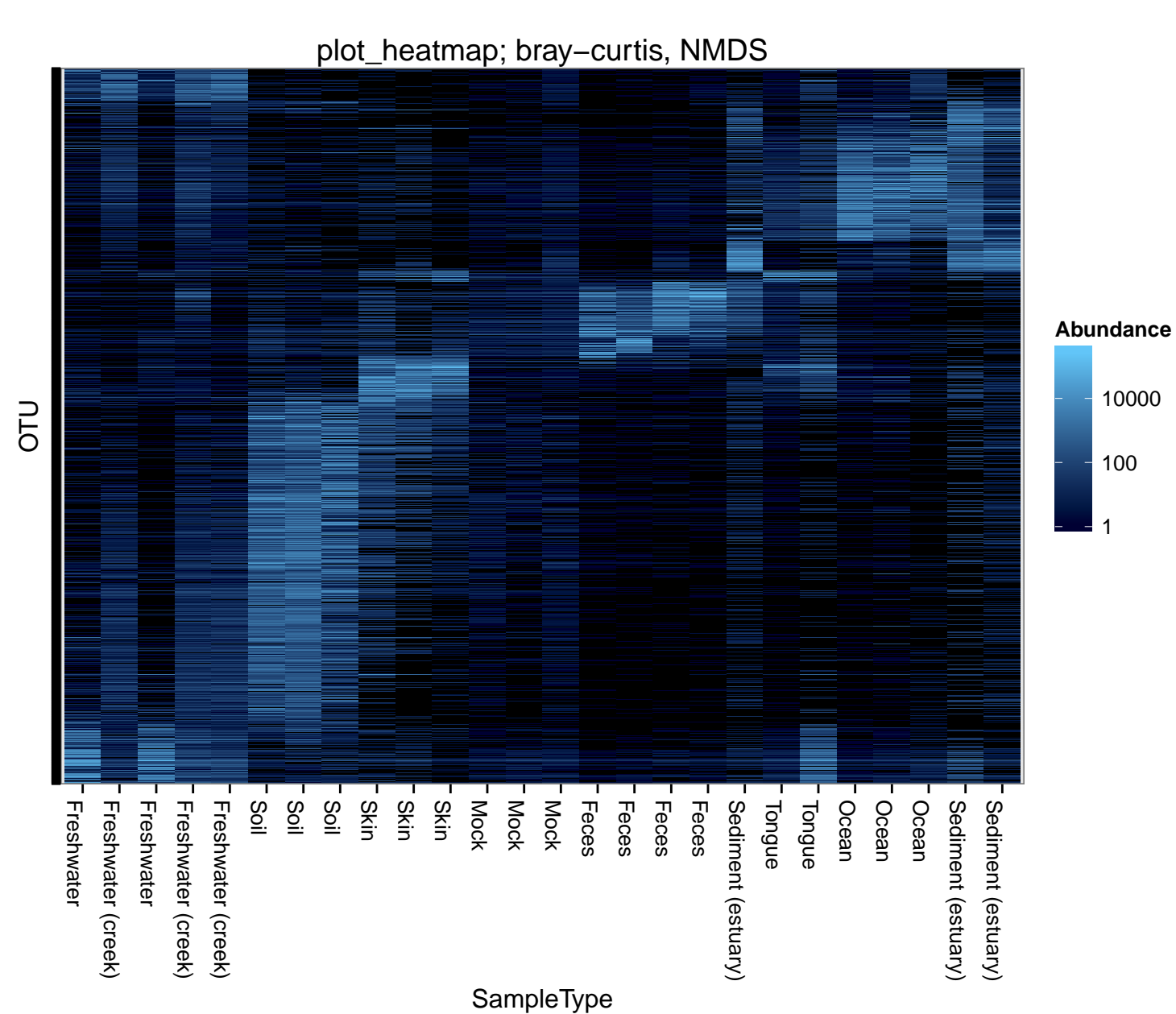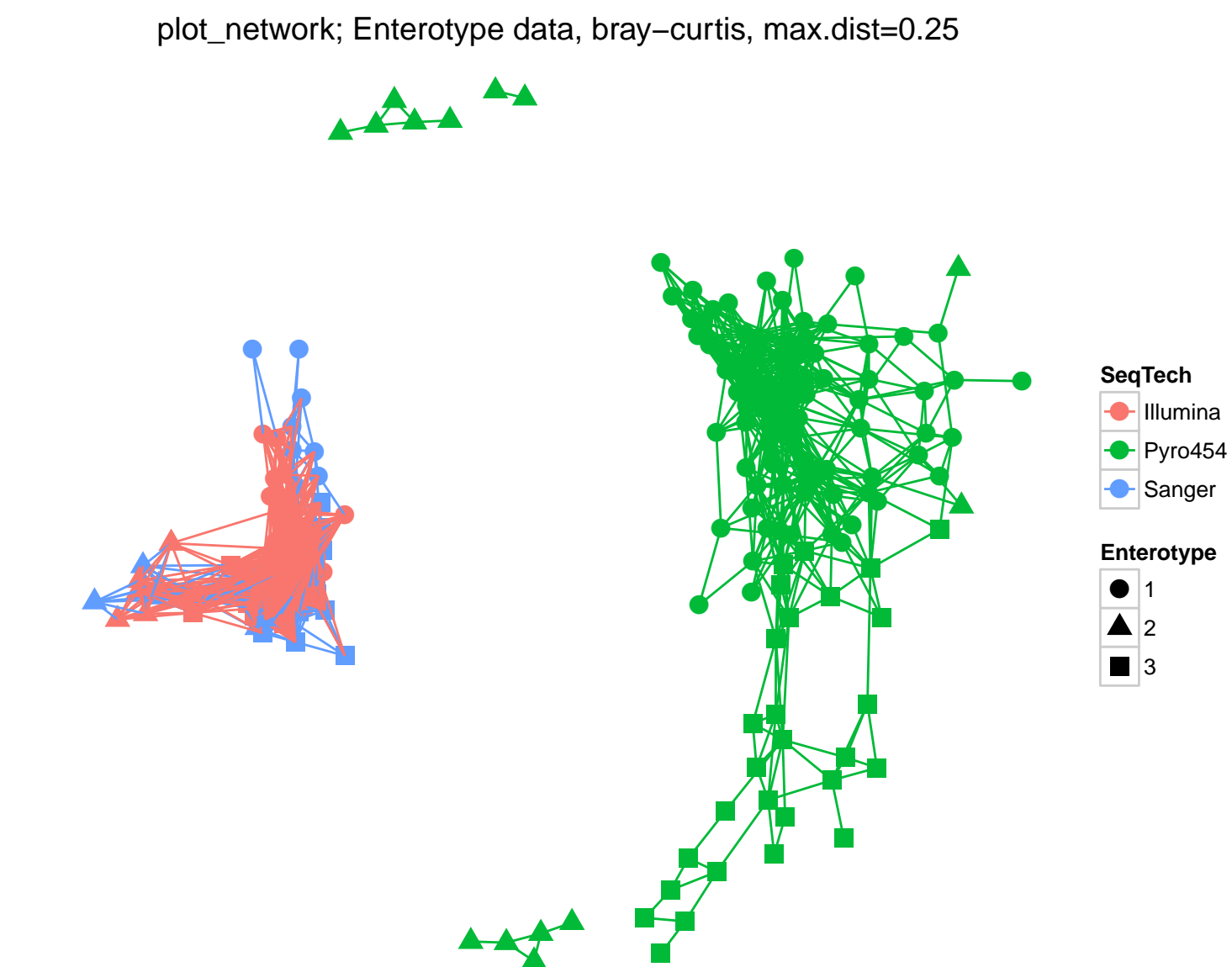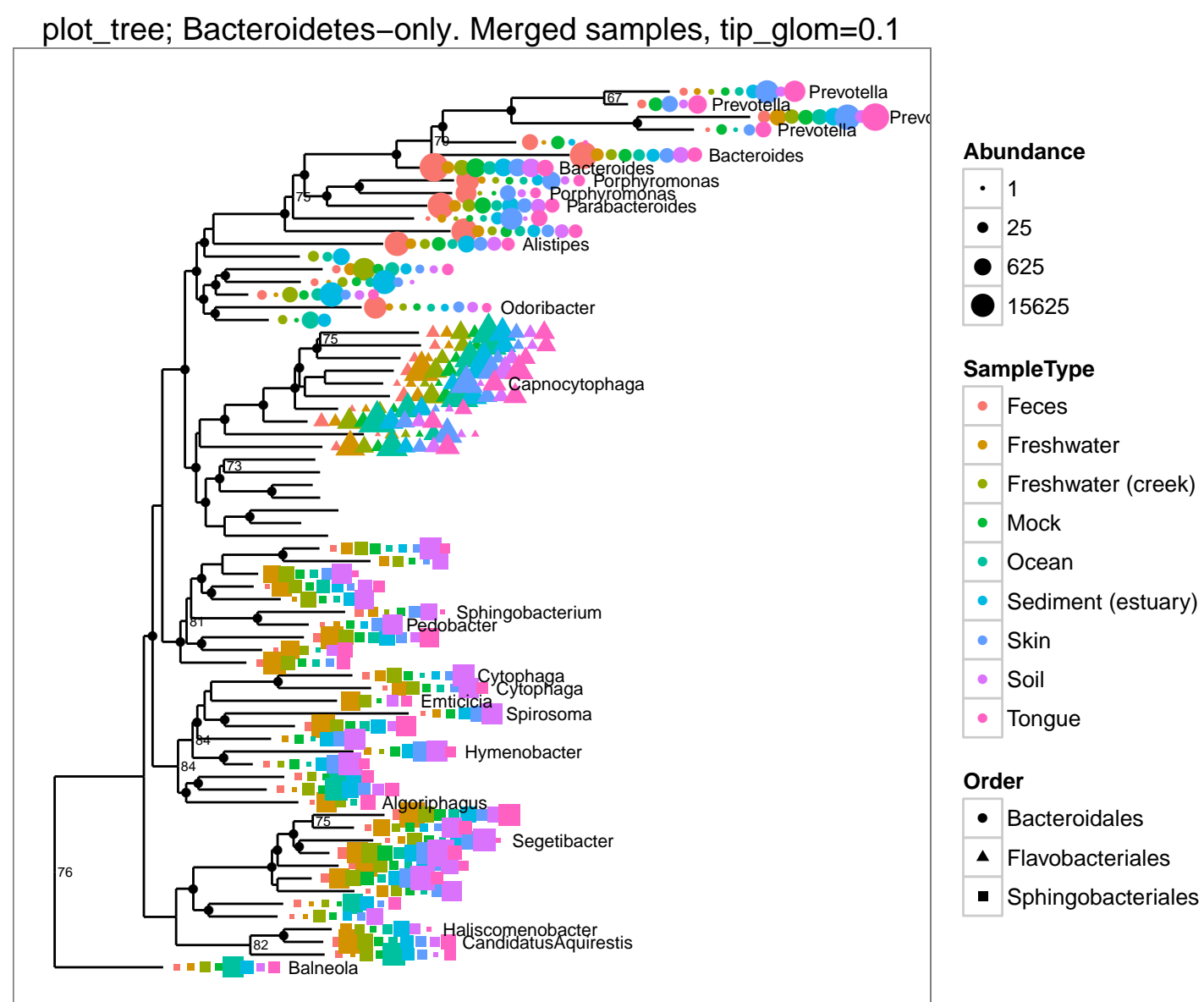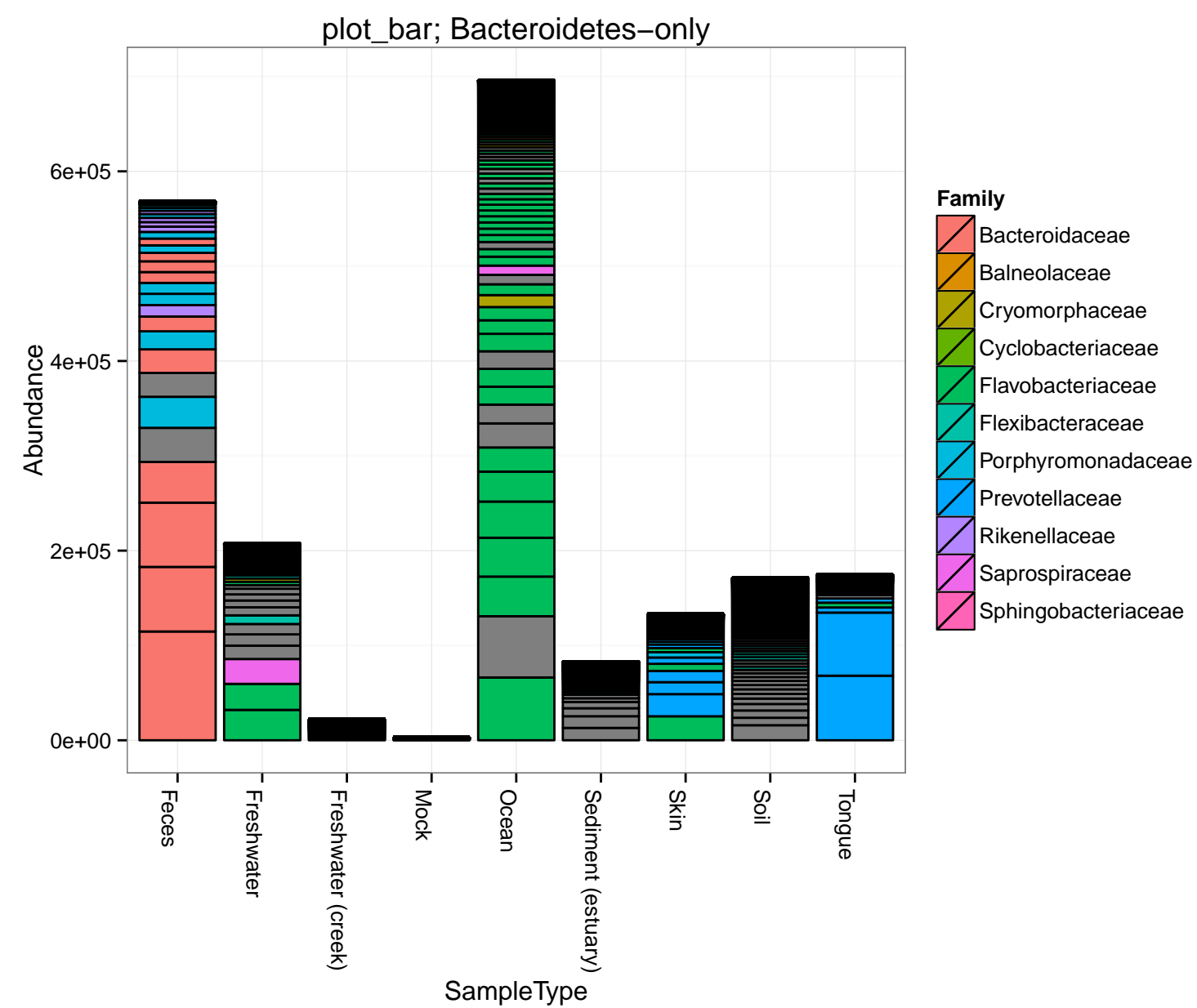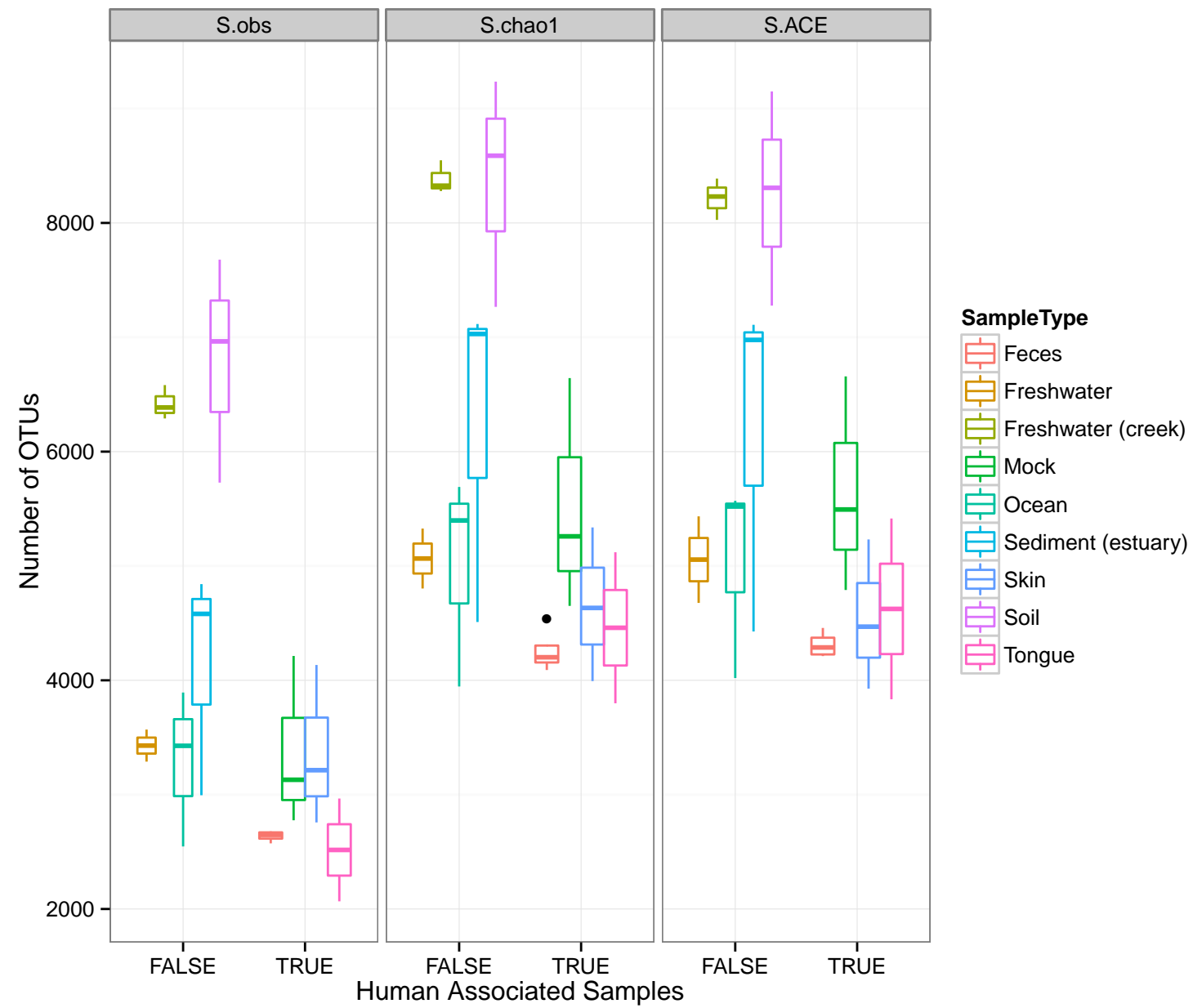

Supplement: File S2 — Source materials for reproducing this manuscript. This is a compressed .zip directory containing the main source file in Sweave .Rnw format [32], as well as the additional files necessary to completely recreate the original manuscript submitted to PLoS ONE. For the uninitiated, Sweave is a R/LaTeX2e interleaved hybrid language format [32] that allows advanced typesetting description to accompany R code and its output (including graphics). Also included is the RFM source file that was used to create Figures 4 and 5, and its accompanying HTML output that includes additional documentation details, links, and intermediate graphics. This latter file is “sourced” (re-run) by the Sweave commands if any of the expected output files are missing. This supporting information zip file also includes R code (at the end of the RFM/HTML files) that demonstrates how to use a phyloseq data object as an argument to other R functions. In this particular example, the bioenv function from the vegan package [92] is demonstrated. (ZIP) [file pone.0061217.s002.zip › phyloseq-article-source-files-figs-code-03/phyloseq-plot-main.pdf]

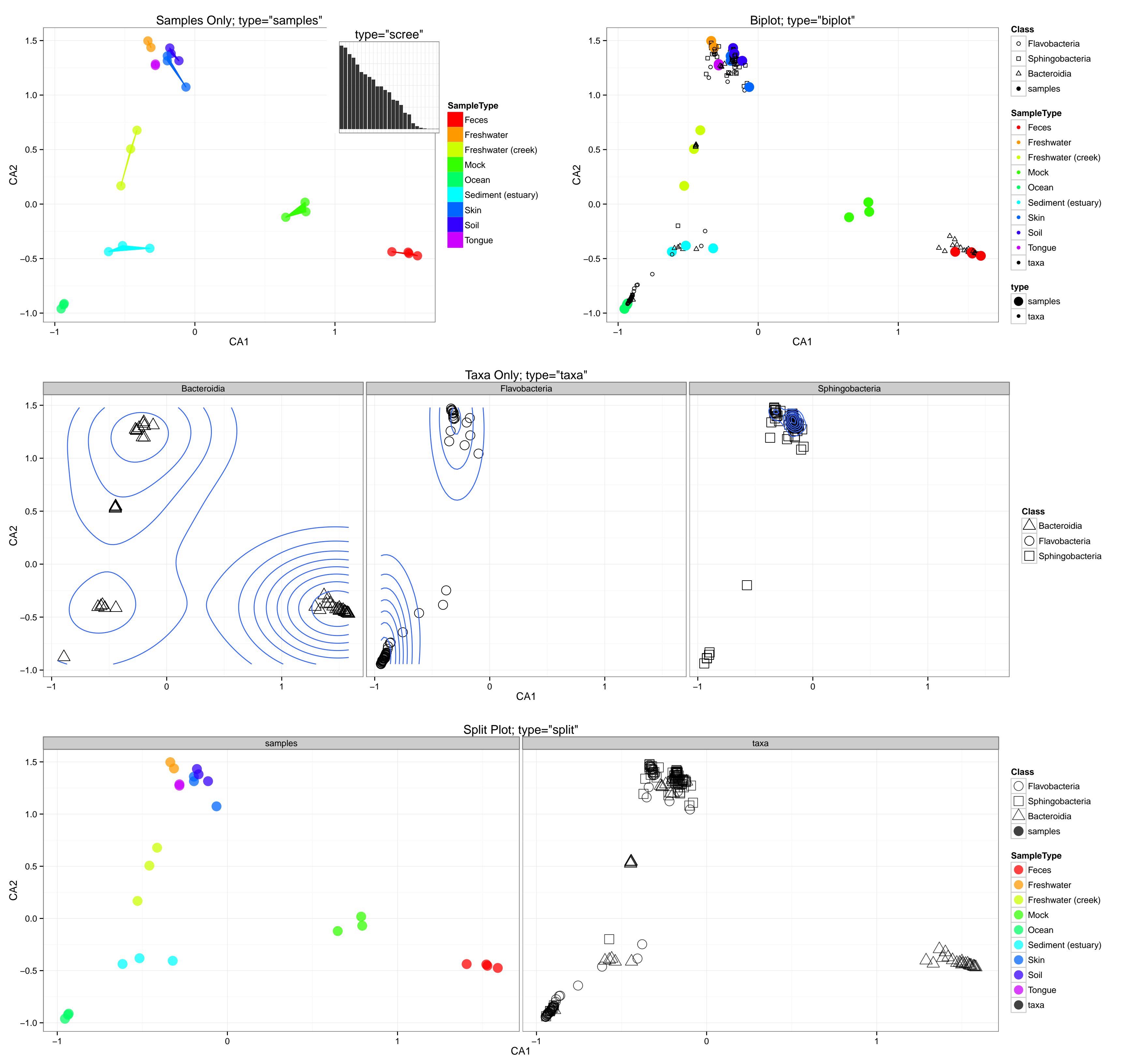

Supplement: File S2 — Source materials for reproducing this manuscript. This is a compressed .zip directory containing the main source file in Sweave .Rnw format [32], as well as the additional files necessary to completely recreate the original manuscript submitted to PLoS ONE. For the uninitiated, Sweave is a R/LaTeX2e interleaved hybrid language format [32] that allows advanced typesetting description to accompany R code and its output (including graphics). Also included is the RFM source file that was used to create Figures 4 and 5, and its accompanying HTML output that includes additional documentation details, links, and intermediate graphics. This latter file is “sourced” (re-run) by the Sweave commands if any of the expected output files are missing. This supporting information zip file also includes R code (at the end of the RFM/HTML files) that demonstrates how to use a phyloseq data object as an argument to other R functions. In this particular example, the bioenv function from the vegan package [92] is demonstrated. (ZIP) [file pone.0061217.s002.zip › phyloseq-article-source-files-figs-code-03/phyloseq-plot-ordination.pdf]

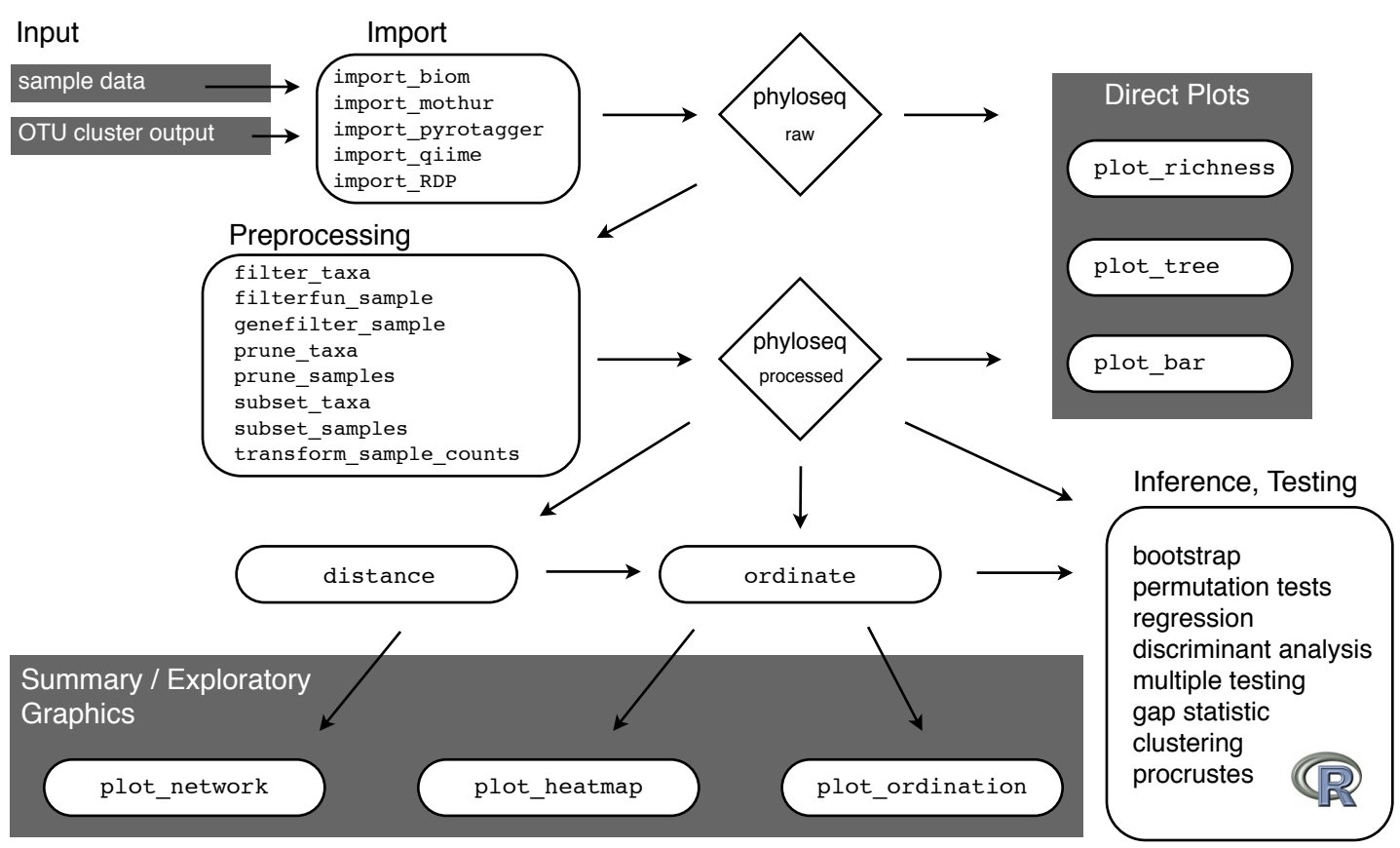

Supplement: File S2 — Source materials for reproducing this manuscript. This is a compressed .zip directory containing the main source file in Sweave .Rnw format [32], as well as the additional files necessary to completely recreate the original manuscript submitted to PLoS ONE. For the uninitiated, Sweave is a R/LaTeX2e interleaved hybrid language format [32] that allows advanced typesetting description to accompany R code and its output (including graphics). Also included is the RFM source file that was used to create Figures 4 and 5, and its accompanying HTML output that includes additional documentation details, links, and intermediate graphics. This latter file is “sourced” (re-run) by the Sweave commands if any of the expected output files are missing. This supporting information zip file also includes R code (at the end of the RFM/HTML files) that demonstrates how to use a phyloseq data object as an argument to other R functions. In this particular example, the bioenv function from the vegan package [92] is demonstrated. (ZIP) [file pone.0061217.s002.zip › phyloseq-article-source-files-figs-code-03/phyloseq-summary-fig.pdf]

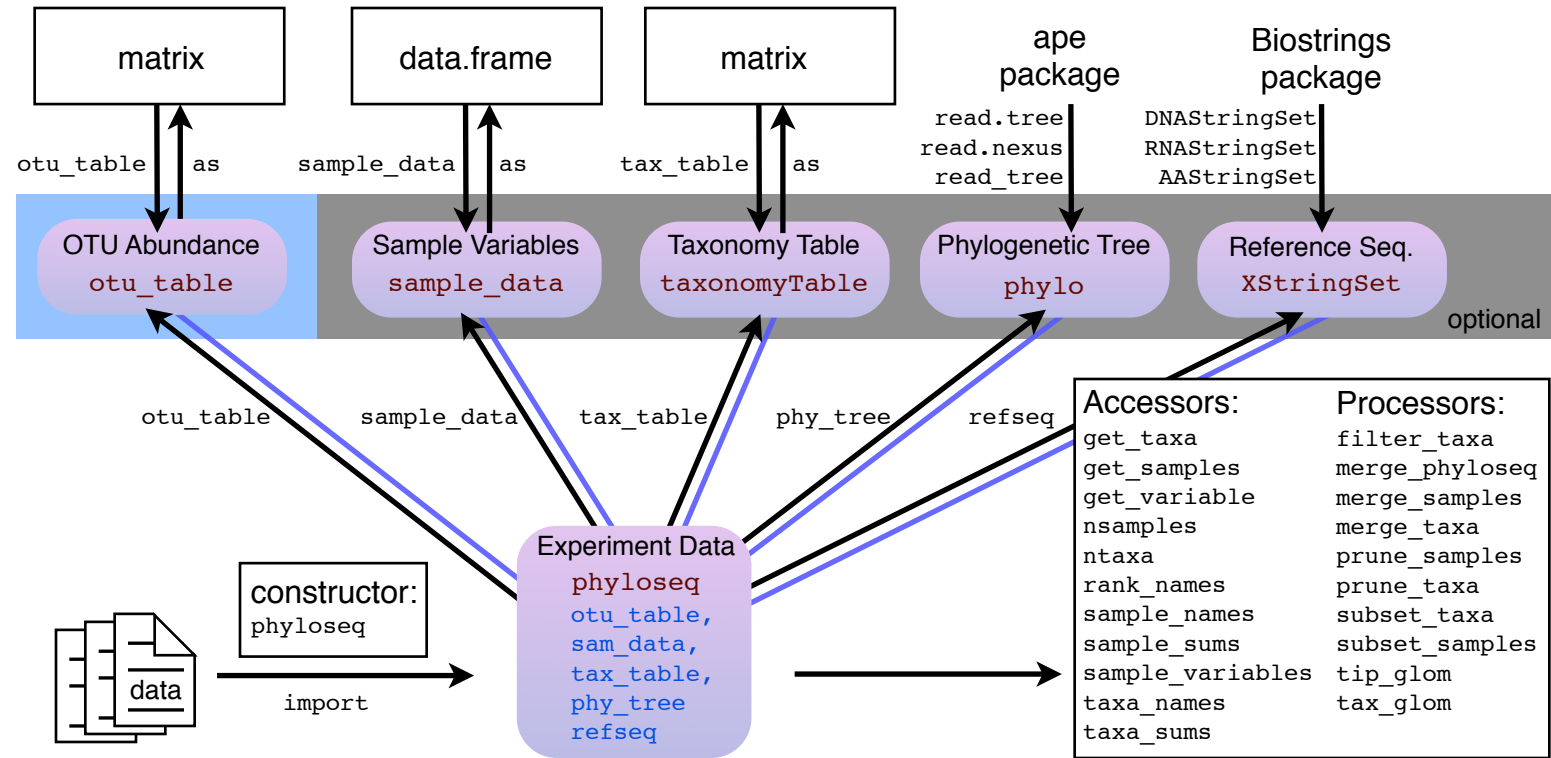

Supplement: File S2 — Source materials for reproducing this manuscript. This is a compressed .zip directory containing the main source file in Sweave .Rnw format [32], as well as the additional files necessary to completely recreate the original manuscript submitted to PLoS ONE. For the uninitiated, Sweave is a R/LaTeX2e interleaved hybrid language format [32] that allows advanced typesetting description to accompany R code and its output (including graphics). Also included is the RFM source file that was used to create Figures 4 and 5, and its accompanying HTML output that includes additional documentation details, links, and intermediate graphics. This latter file is “sourced” (re-run) by the Sweave commands if any of the expected output files are missing. This supporting information zip file also includes R code (at the end of the RFM/HTML files) that demonstrates how to use a phyloseq data object as an argument to other R functions. In this particular example, the bioenv function from the vegan package [92] is demonstrated. (ZIP) [file pone.0061217.s002.zip › phyloseq-article-source-files-figs-code-03/phyloseq_classes_7.pdf]

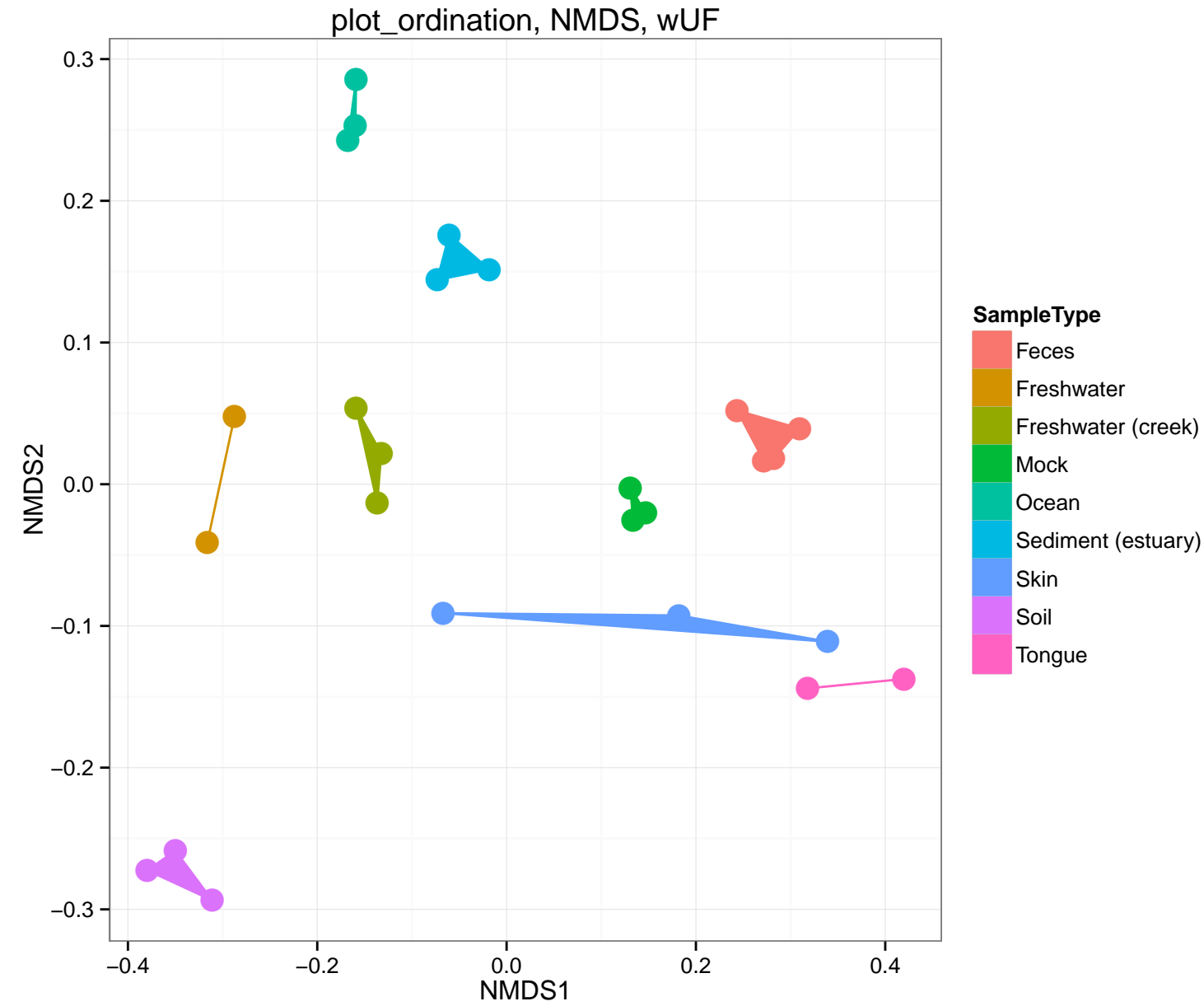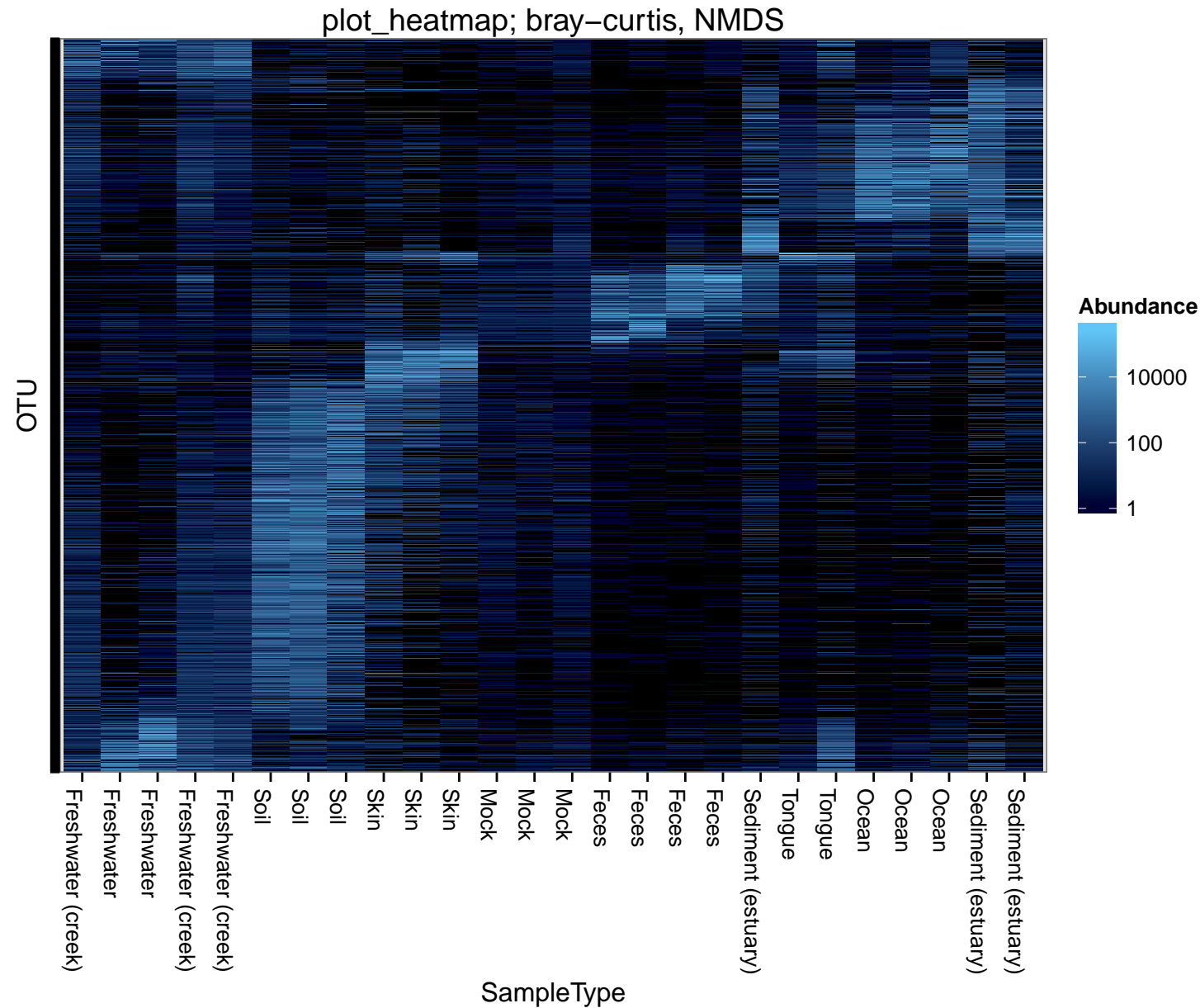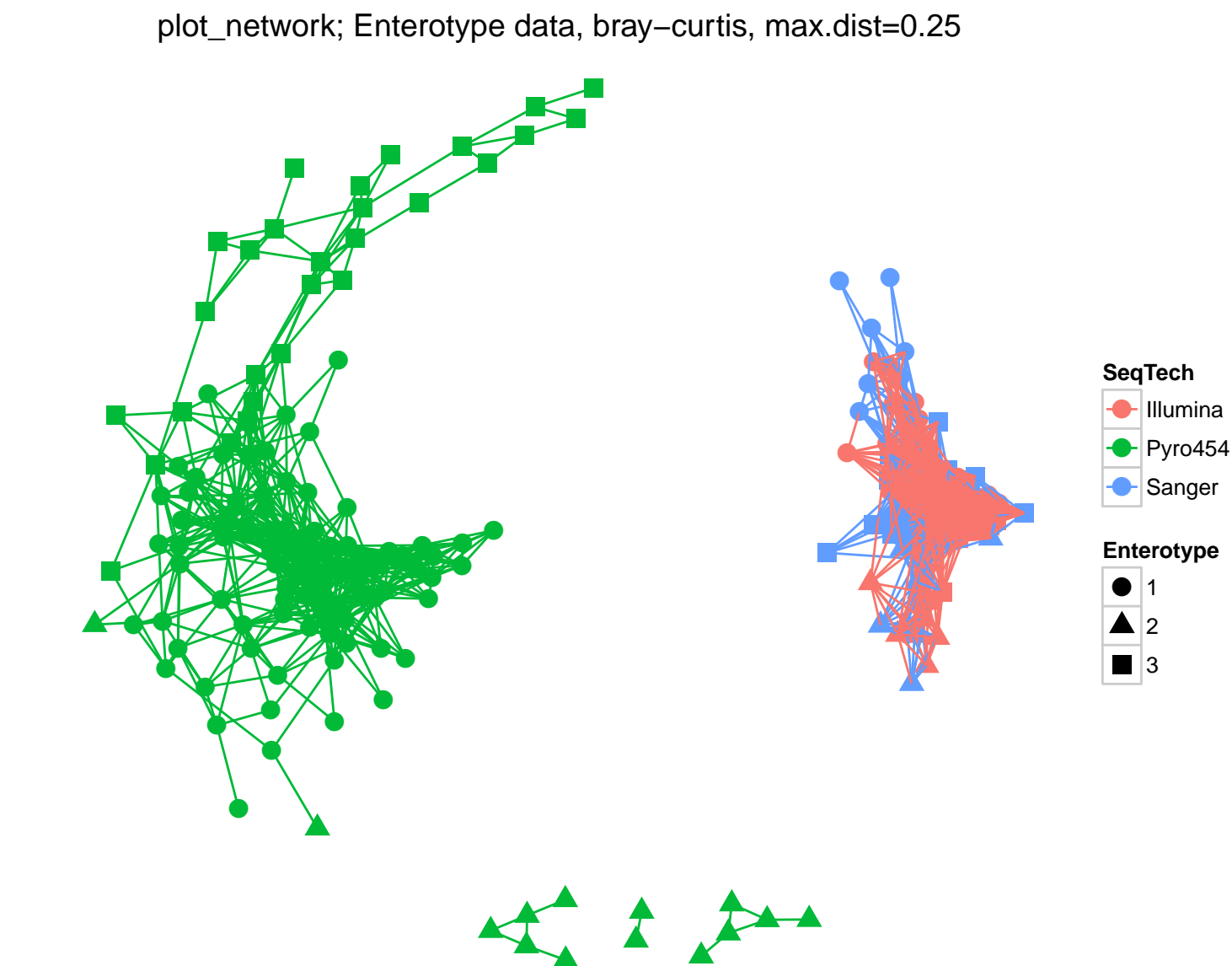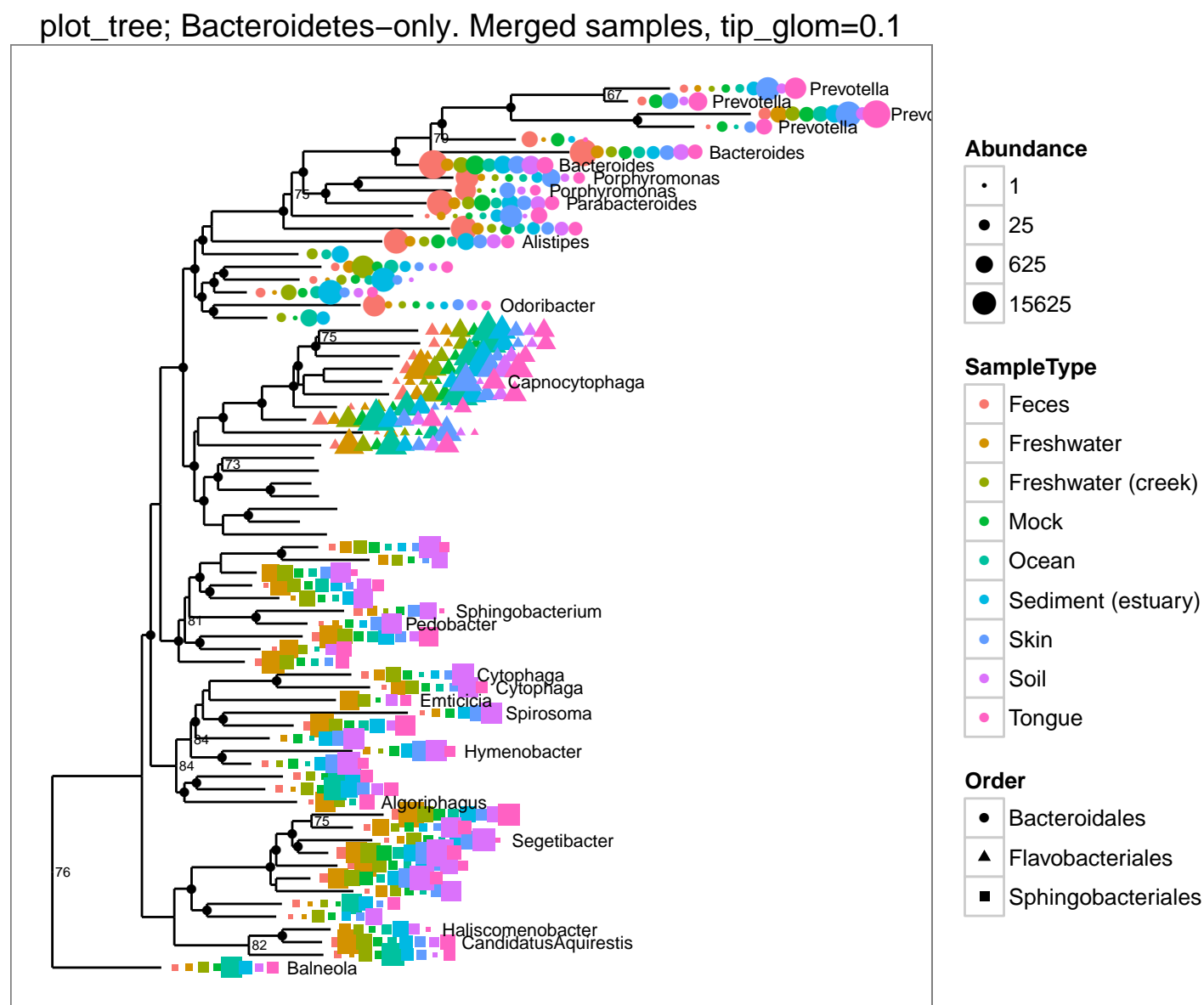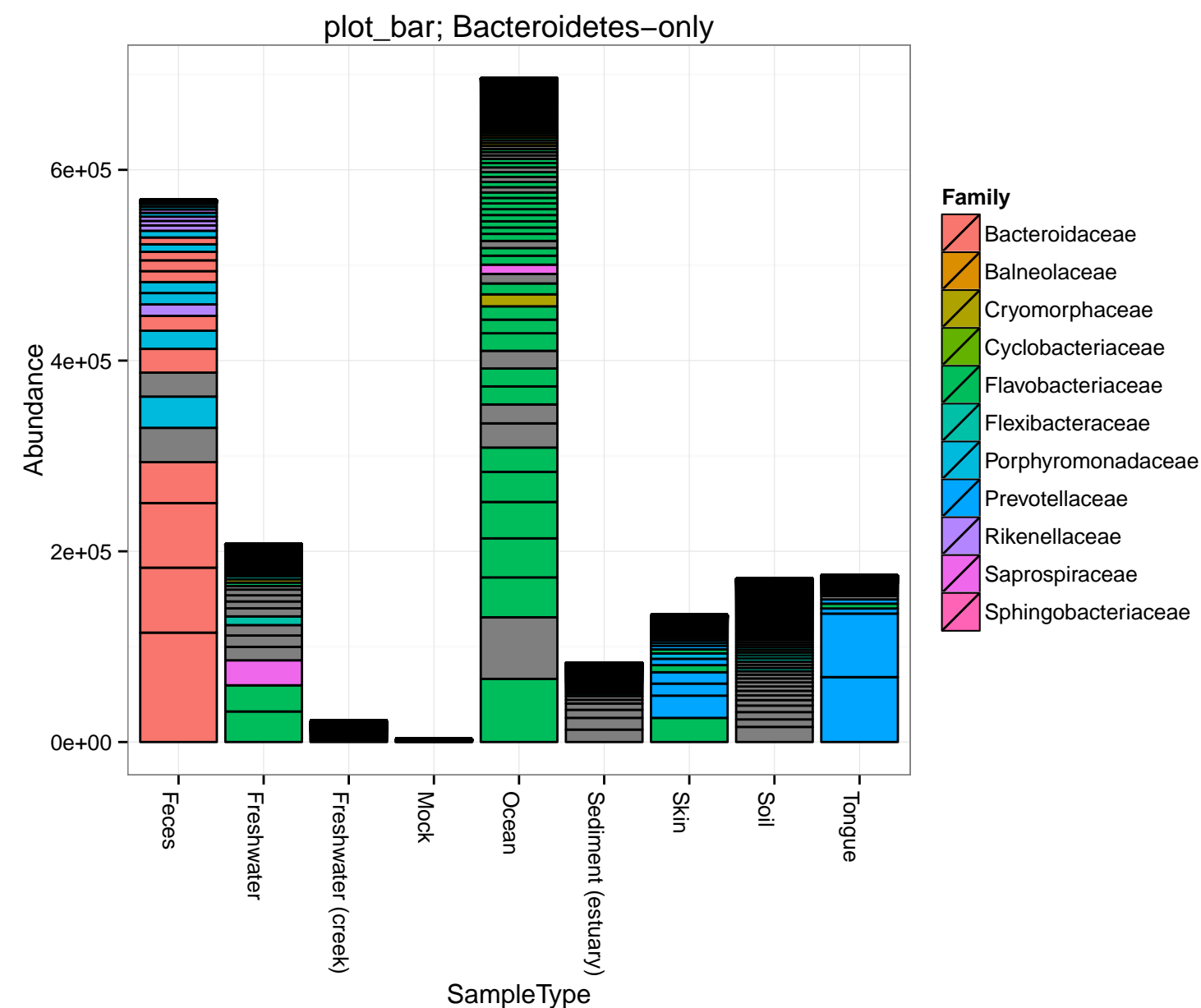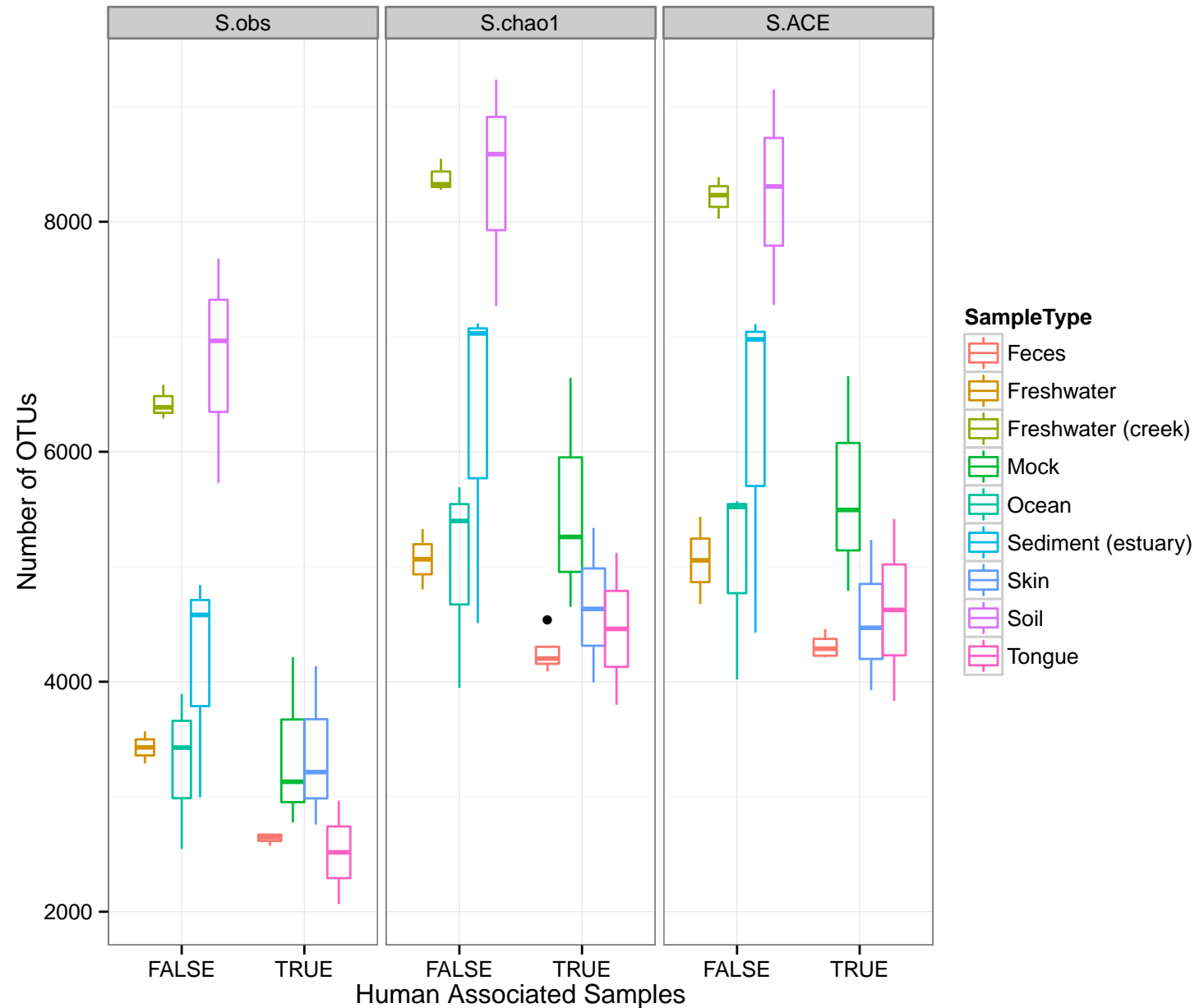

Supplement: File S2 — Source materials for reproducing this manuscript. This is a compressed .zip directory containing the main source file in Sweave .Rnw format [32], as well as the additional files necessary to completely recreate the original manuscript submitted to PLoS ONE. For the uninitiated, Sweave is a R/LaTeX2e interleaved hybrid language format [32] that allows advanced typesetting description to accompany R code and its output (including graphics). Also included is the RFM source file that was used to create Figures 4 and 5, and its accompanying HTML output that includes additional documentation details, links, and intermediate graphics. This latter file is “sourced” (re-run) by the Sweave commands if any of the expected output files are missing. This supporting information zip file also includes R code (at the end of the RFM/HTML files) that demonstrates how to use a phyloseq data object as an argument to other R functions. In this particular example, the bioenv function from the vegan package [92] is demonstrated. (ZIP) [file pone.0061217.s002.zip › phyloseq-article-source-files-figs-code-03/submit-main/phyloseq-plot-main.pdf]

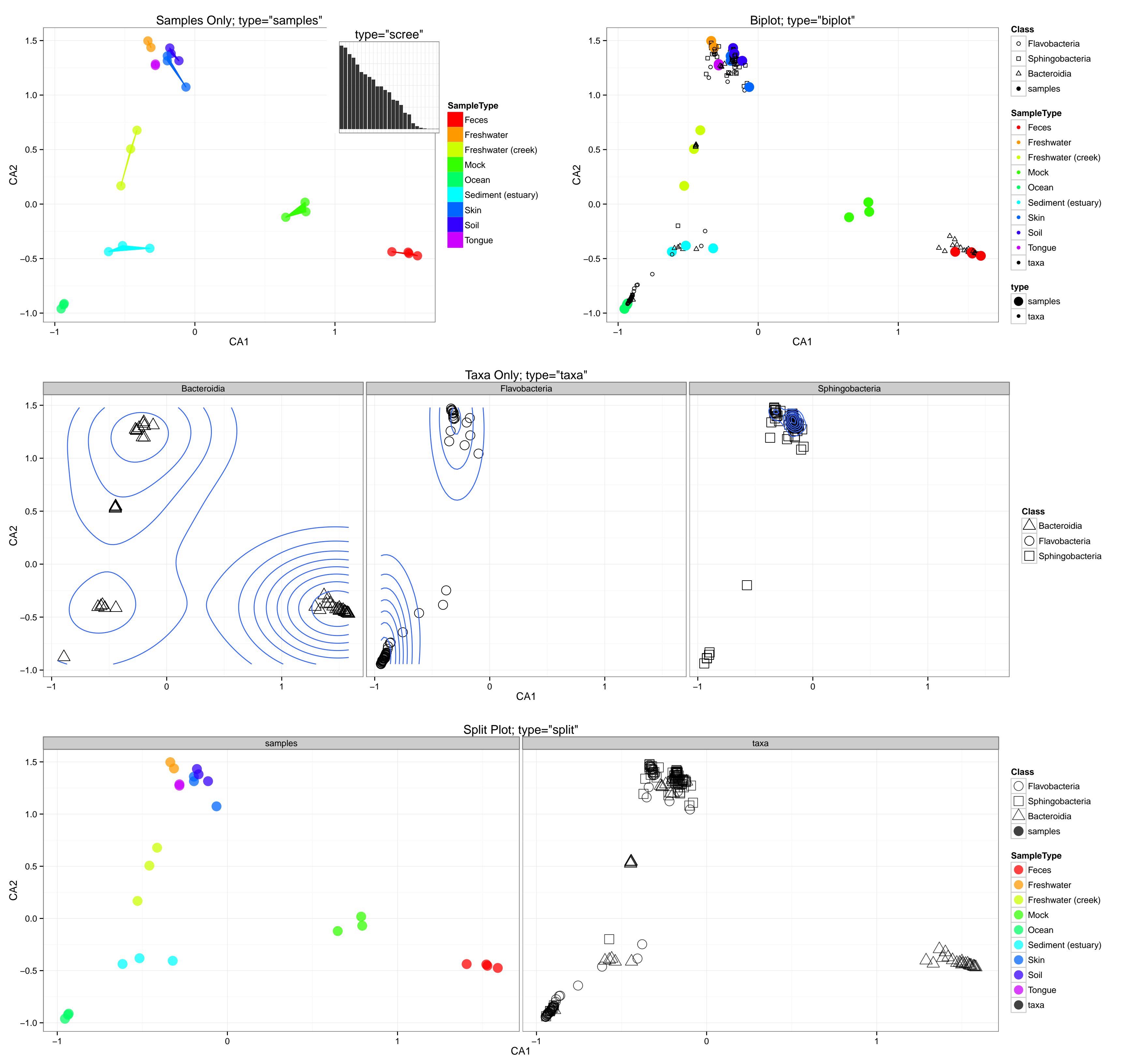

Supplement: File S2 — Source materials for reproducing this manuscript. This is a compressed .zip directory containing the main source file in Sweave .Rnw format [32], as well as the additional files necessary to completely recreate the original manuscript submitted to PLoS ONE. For the uninitiated, Sweave is a R/LaTeX2e interleaved hybrid language format [32] that allows advanced typesetting description to accompany R code and its output (including graphics). Also included is the RFM source file that was used to create Figures 4 and 5, and its accompanying HTML output that includes additional documentation details, links, and intermediate graphics. This latter file is “sourced” (re-run) by the Sweave commands if any of the expected output files are missing. This supporting information zip file also includes R code (at the end of the RFM/HTML files) that demonstrates how to use a phyloseq data object as an argument to other R functions. In this particular example, the bioenv function from the vegan package [92] is demonstrated. (ZIP) [file pone.0061217.s002.zip › phyloseq-article-source-files-figs-code-03/submit-main/phyloseq-plot-ordination.pdf]
